# Efficacy and Safety of Cariprazine, Asenapine, Xanomeline–Trospium, and Lumateperone for Acute Exacerbations of Schizophrenia in Adults: A Network Meta-Analysis

**DOI:** 10.1093/schizbullopen/sgaf024

**Published:** 2025-10-16

**Authors:** Alaaeddin Abusalameh, Celina R Andonie, Mahmoud Ladadweh, Tamer Hodrob, Ibrahim Ismail, Hazem Ayesh

**Affiliations:** Faculty of Medicine, Al-Quds University, East Jerusalem, 91105, Palestine; Faculty of Medicine, Al-Quds University, East Jerusalem, 91105, Palestine; Faculty of Medicine, Al-Quds University, East Jerusalem, 91105, Palestine; Faculty of Medicine, Al-Quds University, East Jerusalem, 91105, Palestine; Faculty of Medicine, Al-Quds University, East Jerusalem, 91105, Palestine; Deaconess Clinic Endocrinology, Deaconess Health System, Evansville, IN 47708, United States

**Keywords:** schizophrenia, PANSS total score, cariprazine, lumateperone, asenapine, xanomeline–trospium

## Abstract

**Objective:**

This network meta-analysis evaluated the efficacy and safety of cariprazine, asenapine, xanomeline–trospium chloride, and lumateperone in treating acute exacerbations of schizophrenia in adults, compared with placebo.

**Methods:**

A systematic search of PubMed, Scopus, Cochrane Central, and ClinicalTrials.gov identified 12 randomized controlled trials (RCTs; *n* = 4584 patients). Efficacy was assessed using changes in Positive and Negative Syndrome Scale (PANSS) total scores; safety outcomes included adverse events (AEs), treatment discontinuation, and specific side effects.

**Results:**

Asenapine (MD = −9.83; 95% CI, −13.49 to −6.18), xanomeline–trospium (MD = −9.80; 95% CI, −13.00 to −6.60), and cariprazine (MD = −8.41; 95% CI, −11.33 to −5.50) showed statistically significant PANSS score reductions. Lumateperone, however, did not reach statistical significance (MD = −2.79; 95% CI, −6.60 to 1.01). Safety profiles varied: cariprazine had the lowest risk of serious AEs (RR = 0.52), while xanomeline–trospium was associated with gastrointestinal AEs (RR = 3.86), and asenapine with weight gain (RR = 3.12).

**Conclusions:**

This network meta-analysis found that among asenapine, xanomeline–trospium, lumateperone and cariprazine, asenapine, xanomeline–trospium, and cariprazine were effective in reducing PANSS total score compared with placebo, whereas lumateperone did not show statistically significant reduction compared with placebo. These findings offer important insights into efficacy and safety of these 4 medications in the treatment of acute exacerbation of schizophrenia. However, further head-to-head comparisons with standard treatments are needed to guide a more evidence-based selection. Future trials with longer durations and more diverse populations are warranted to confirm and extend these findings.

## Introduction

Schizophrenia is a mental disorder characterized by disruptions in cognition, perceptions, emotions, and social functioning.^1^^,^^2^ Acute exacerbation refers to a sudden worsening of symptoms of schizophrenia, which include positive symptoms (hallucinations, delusions, and thought disorder) as well as negative symptoms (reduced expression of emotions, reduced motivation to accomplish goals), cognitive deficits, and impaired social and occupational functioning.^2^ While precise prevalence estimates are challenging due to methodological and diagnostic variability, schizophrenia and related psychotic disorders are estimated to affect approximately 0.25% and 0.64% in the United States^3-5^ and 0.33%-0.75% internationally.^6^^,^^7^ Despite its low prevalence, schizophrenia ranks among the top 15 causes of disability worldwide and is associated with significant morbidity, elevated risk of comorbid psychiatric conditions, and increased premature mortality.^8^ Although no curative treatment exists, first- and second-generation antipsychotics remain the mainstay of management.^9^

While antipsychotics remain the cornerstone of acute exacerbation of schizophrenia management,^9^ several pharmacologic options—particularly cariprazine, asenapine, xanomeline–trospium chloride, and lumateperone—have demonstrated promising results in improving symptoms as measured by Positive and Negative Syndrome Scale (PANSS) total score with a favorable safety profile.^10-16^ Interestingly, cariprazine, asenapine, and lumateperone target dopamine receptors,^17-19^while xanomeline–trospium chloride targets solely muscarinic receptors, a relatively novel mechanism that may represent a significant therapeutic advancement in the field.^20^^,^^21^ Individual randomized controlled trials (RCTs) have reported variable outcomes, and no single agent has emerged as the preferred treatment. Prior pairwise meta-analyses have provided limited insight due to a lack of direct head-to-head comparisons between these 4 medications.

This meta-analysis aims to evaluate the efficacy of cariprazine, asenapine, xanomeline–trospium chloride, and lumateperone in the treatment of acute exacerbations of schizophrenia in adults compared with placebo. We focus on key outcomes such as change in PANSS total score and adverse events. The analysis includes only RCTs and synthesizes direct and indirect evidence through a frequentist network meta-analysis framework.

To date, no comprehensive network meta-analysis has directly compared cariprazine, asenapine, xanomeline–trospium chloride, and lumateperone, leaving clinicians with limited evidence to inform comparative effectiveness and contributing to uncertainty in treatment selection during acute exacerbation of schizophrenia. A network meta-analysis enables the integration of both direct and indirect evidence across multiple interventions. This approach facilitates treatment ranking and provides clinicians with a clearer understanding of how cariprazine, asenapine, xanomeline–trospium chloride, and lumateperone compares within the broader therapeutic landscape.

## Methods

This systematic review and network meta-analysis was conducted in accordance with the PRISMA-NMA guidelines.^22^ The protocol was prospectively registered on the Open Science Framework (OSF; Registration DOI: 10.17605/OSF.IO/ZV2M6).^23^ The study design incorporated a frequentist framework using the netmeta R package to synthesize direct and indirect evidence from RCTs.^24^

### Study Selection

We included RCTs assessing pharmacologic therapies in adult patients with acute exacerbations of schizophrenia, as defined by DSM-IV or DSM-5 criteria. Eligible interventions included cariprazine, asenapine, xanomeline–trospium chloride, or lumateperone. Comparators included placebo or other active drugs. Eligible studies were required to report primary efficacy outcome measured by the PANSS total score. Studies were excluded if they focused on pediatric populations, non-pharmacologic interventions, patients diagnosed with other psychiatric conditions, did not report relevant outcomes, or were non-comparative studies or conference abstracts without full-text availability. Post-hoc analysis and extension studies were excluded (Supplementary  Material S3).

### Search Strategy

A comprehensive literature search was conducted across PubMed, Scopus, the Cochrane Central Register of Controlled Trials, and Clinical Trials.gov from database inception to February 2025. Search terms included combinations of (“schizophrenia” OR “psychosis” OR “acute psychosis” OR “schizophrenia exacerbation” OR “acute phase schizophrenia” OR “psychotic episode” OR “acute psychiatric decompensation”) AND (“Cariprazine” OR “Vraylar” OR “Asenapine” OR “Saphris” OR “Secuado” OR “Xanomeline-Trospium chloride” OR “KarXT” OR “Lumateperone” OR “Caplyta”) AND (“randomized controlled trial” OR RCT OR “clinical trial” OR “trial”) AND (“PANSS” OR “PANSS positive subscale” OR “Simpson Angus” OR “adverse events” OR safety). The detailed search strategy is reported in Supplementary Material S1. No restrictions were applied based on publication status; however, only studies published in English or with available English translations were included.

### Screening Process

Two authors (A.A. and M.L.) independently screened titles and abstracts, followed by full-text reviews to identify eligible studies. Discrepancies were resolved by consensus or by a third author (C.A.).

### Data Extraction

Two independent reviewers extracted data using a standardized and piloted form. Collected variables included study characteristics (eg, sample size, duration, and region), patient demographics, intervention details, and outcome measures. Specifically, data on age, sex, body mass index, race, PANSS total score, and Clinical Global Impressions-Severity of illness score (CGI-S) were collected (Supplementary Materials S3 and S4). Combined means and standard deviations were calculated following the Cochrane Handbook for Systematic Reviews of Interventions guidelines.^25^

### Statistical Analysis

We conducted a frequentist random-effects network meta-analysis using the netmeta package in R to compare multiple treatments by analyzing data from various studies, allowing for both direct and indirect comparisons.^24^ This method synthesizes evidence to determine the relative effectiveness of each treatment, even if some treatments were not directly compared in any individual study.^26^ Relative Risks (RRs) were used for dichotomous outcomes, and mean differences (MDs) were used for continuous outcomes, each with 95% confidence intervals. Placebo was used as the reference comparator. When multiple doses were available for an intervention, the highest tolerated dose was used for primary analysis. Treatment rankings were estimated using *P*-scores derived from the netrank function with higher *P*-scores associated with better outcomes.^27^ Heterogeneity across studies was assessed using the *I*^2^ statistic, tau,^2^ and Cochran’s *Q* test.^28^ Global inconsistency was evaluated via design-by-treatment interaction models using the decomp.design() function. We assessed transitivity by comparing baseline covariates (eg, age, gender) across treatment comparisons using ANOVA and boxplot visualizations.^29^ Publication bias was assessed using funnel plots and Egger’s test for the primary outcomes (Supplementary Material S6). Sensitivity analyses included exclusion of high-risk bias studies, exclusion of small sample size studies and leave-one-out analyses to evaluate the influence of individual trials on overall estimates. The evaluated outcome is PANSS total score. Safety outcomes include serious adverse events, treatment discontinuation due to treatment-emergent adverse events, and the incidence of gastrointestinal symptoms, akathisia, sedation and/or somnolence, and ≥ 7 increase in body weight among participants (Supplementary Material S10).

### Bias Assessment and Certainty of Evidence

The Cochrane Risk of Bias (ROB2) tool was used to assess study quality, with each domain scored as 1 (low), 2 (moderate), or 3 (high) risk.^30^ Two authors (A.A. and C.A.) independently assessed the risk of bias. Disagreements were resolved through discussion or by consulting a third author (M.L.). We also evaluated overall confidence in network estimates using the CINeMA (Confidence in Network Meta-Analysis) framework^31^ (Supplementary Material S5).

## Results

### Study Characteristics

**Figure 1:**
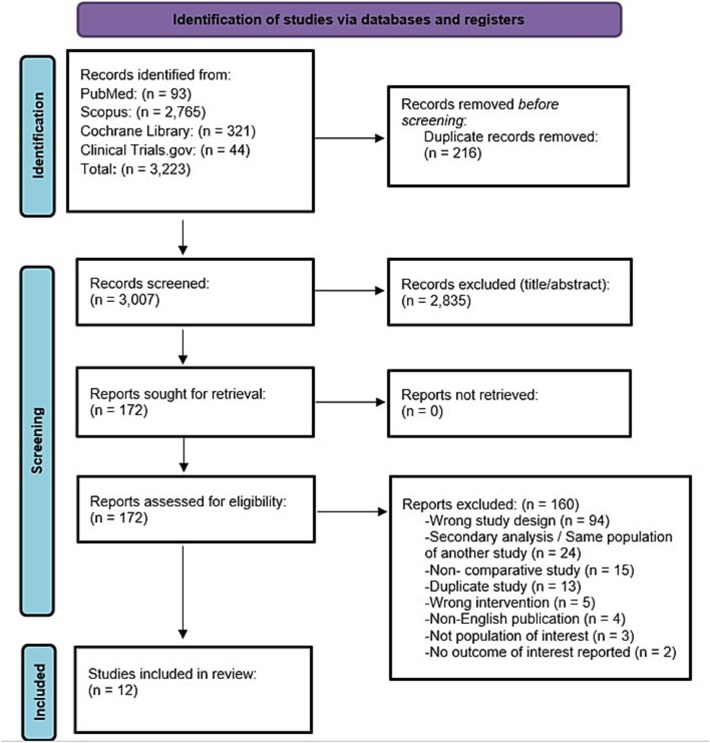
PRISMA Flowchart for Study Selection

**Figure 2:**
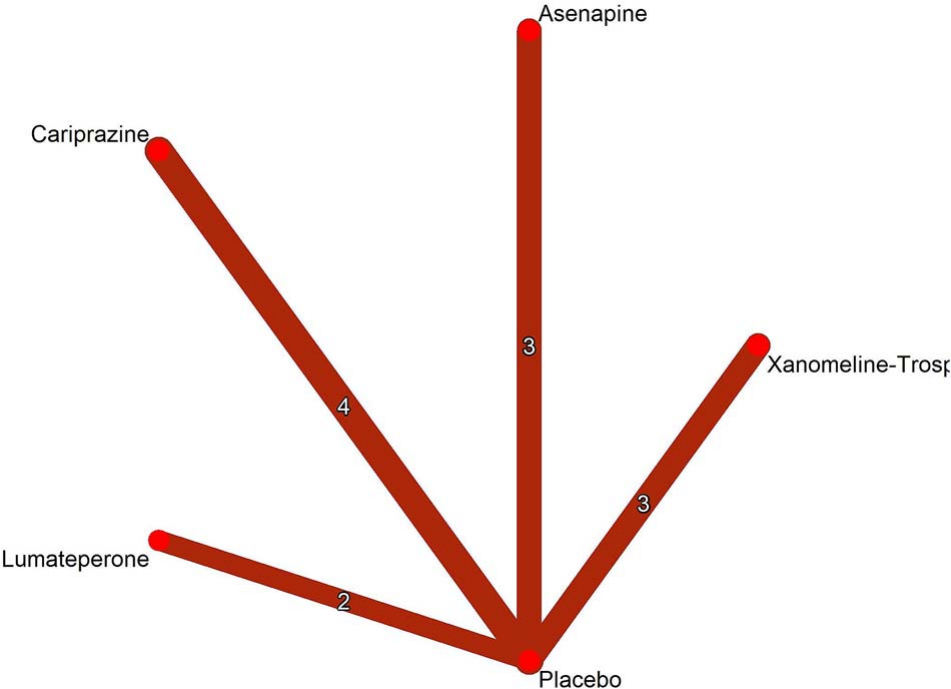
Network plot of treatment comparisons for PANSS Total Score. This network plot shows the direct comparisons among cariprazine, asenapine, xanomeline–trospium chloride, lumateperone, and placebo in studies for PANSS total score. Edge thickness indicates the number of direct comparisons between treatments

A total of 3223 records were identified through database searching, of which 172 full-text articles were assessed for eligibility. Twelve RCTs (Table 1; Figure 1), encompassing 4584 patients, were included in the analysis.^15^^,^^16^^,^^32-41^ These studies compared cariprazine, asenapine, xanomeline–trospium chloride, or lumateperone, and placebo. Study durations ranged from 4 to 6 weeks. The network for the included interventions is presented in Figure 2. All trials enrolled adult patients with acute exacerbations of schizophrenia, as defined by DSM-IV or DSM-5 criteria. The mean age of participants was 40.68 years (SD 10.62). The mean of baseline PANSS total score was 94.60 (SD 10.73). Baseline characteristics are summarized in Table 2. The risk of bias was generally rated as some concern, with some domains having low risk (Supplementary Material S5). Publication bias was generally rated as low concern (Supplementary Material S6). The certainty of evidence was generally low due to concerns of reporting bias, imprecision, heterogeneity, and within-study bias (Supplementary Material S9). Baseline characteristics of the included studies and the participants are summarized in Supplementary  Materials S3 and S4.

**Table 1:** Study Characteristics and Outcomes in the Included Clinical Trials

**Study**	**Design**	**Registration**	**Duration**	**Geographic location**	**Treatment arms**	**Number of participants**	**Primary outcomes**	**Population**
Kinoshita (2016)^32^	Phase III	NCT01098110	6 weeks	Japan, Korea, and Taiwan	Asenapine (5 mg, 10 mg twice daily), placebo	532	PANSS total score	Asian patients 20-64 years with schizophrenia, exacerbation of ≤ 2 months, PANSS total score ≥ 60, CGI-S rating ≥ 4.
Landbloom (2016)^33^	Phase III	NCT01617187	6 weeks	United States, Bulgaria, Romania, Russian Federation, Croatia, and Ukraine	Asenapine (2.5 mg, 5 mg twice daily), olanzapine (15 mg once daily), placebo	360	PANSS total score	Patients (≥18 years) with schizophrenia, exacerbation of ≤ 8 weeks, PANSS total score ≥ 70, CGI-S rating ≥ 4.
Potkin (2007)^34^	NR	NR	6 weeks	United States	Asenapine (5 mg twice daily), risperidone (3 mg twice daily), placebo	182	PANSS total score	Patients (≥18 years) with schizophrenia, PANSS total score ≥ 60, CGI-S rating ≥ 4.
Durgam (2014)^35^	Phase II	NCT00694707	6 weeks	United States, India, Russia, Ukraine, and Malaysia	Cariprazine (1.5 mg daily), cariprazine (3 mg daily), cariprazine (4.5 mg daily), risperidone (4 mg daily), placebo	732	PANSS total score	Patients 18-60 years with schizophrenia, exacerbation of ≤ 2 weeks, PANSS total score between 80 and 120, CGI-S rating ≥ 4.
Durgam (2015)^36^	Phase III	NCT01104766	6 weeks	United States, Romania, Russia, and Ukraine	Cariprazine (3 mg daily), cariprazine (6 mg daily), aripiprazole (10 mg daily), placebo	465	PANSS total score	Patients 18-60 years with schizophrenia, exacerbation of ≤ 2 weeks, PANSS total score between 80 and 120, CGI-S rating ≥ 4.
Durgam (2016)^37^	Phase II	NCT00404573	6 weeks	United States	Cariprazine (1.5–4.5 mg daily), cariprazine (6–12 mg daily), placebo	392	PANSS total score	Patients 18-65 years with schizophrenia, exacerbation of ≤ 2 weeks, PANSS total score between 80 and 120, CGI-S rating ≥ 4.
Kane (2015)^38^	Phase III	NCT01104779	6 weeks	United States, India, Colombia, and South Africa	Cariprazine (3-6 mg daily), cariprazine (6–9 mg daily), placebo	446	PANSS total score	Patients 18-60 years with schizophrenia, exacerbation of ≤ 2 weeks, PANSS total score between 80 and 120, CGI-S rating ≥ 4.
Correll (2020)^15^	Phase III	NCT02282761	4 weeks	United States	Lumateperone (28 mg daily), lumateperone (42 mg daily), placebo	450	PANSS total score	Patients 18-60 years with schizophrenia, exacerbation of ≤ 4 weeks, PANSS total score ≥ 70, CGI-S rating ≥ 4, BPRS total score ≥ 40.
Lieberman (2016)^16^	Phase II	NCT01499563	4 weeks	United States	Lumateperone (60 mg daily), lumateperone (120 mg daily), risperidone (4 mg daily), placebo	335	PANSS total score	Patients 18-60 years with schizophrenia, exacerbation of ≤ 4 weeks, BPRS total score ≥ 40.
Brannan (2021)^41^	Phase II	NCT03697252	5 weeks	United States	Xanomeline–trospium (50-125 mg—20-30 mg twice daily), placebo	182	PANSS total score	Patients 18-60 years with schizophrenia, exacerbation of ≤ 2 months, PANSS total score between 80 and 120, CGI-S score ≥ 4.
Kaul (2024_a)^39^	Phase III	NCT04659161	5 weeks	United States	Xanomeline–trospium (50-125 mg—20-30 mg twice daily), placebo	252	PANSS total score	Patients 18-65 years with schizophrenia, exacerbation of ≤ 2 months, PANSS total score between 80 and 120, CGI-S score ≥ 4.
Kaul (2024_b)^40^	Phase III	NCT04738123	5 weeks	United States and Ukraine	Xanomeline–trospium (50-125 mg—20-30 mg twice daily), placebo	256	PANSS total score	Patients 18-65 years with schizophrenia, exacerbation of ≤ 2 months, PANSS total score between 80 and 120, CGI-S score ≥ 4.

Abbreviations: PANSS, Positive and Negative Syndrome Scale; CGI-S, Clinical Global Impressions-Severity of illness; BPRS, Brief Psychiatric Rating Scale; NR, Not Reported.

The table summarizes various clinical trials, detailing study design, registration, duration, treatment arms, primary and secondary outcomes, and population characteristics.

### PANSS Total Score

**Figure 3:**
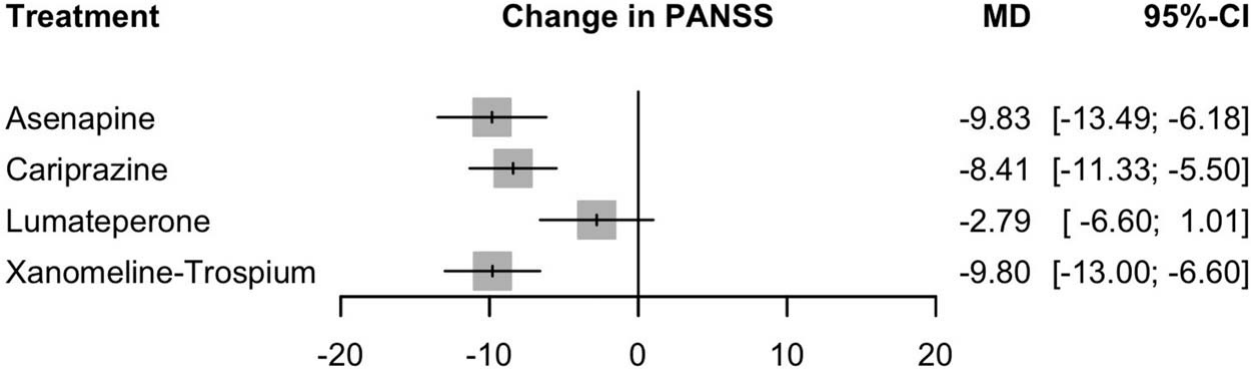
Forest plot comparing the efficacy of cariprazine, asenapine, xanomeline–trospium chloride, and lumateperone treatments against placebo in patients with acute exacerbation of schizophrenia. The outcome measured is PANSS total score. The mean difference (MD) with 95% confidence intervals (CI) shows the likelihood of PANSS total improvement for each treatment compared with placebo. Grey squares represent effect estimates; horizontal lines show 95% CIs

In the random effects model assessing the improvement in PANSS total score, asenapine (MD = −9.83; 95% CI: −13.49 to −6.18; *P* < .0001), xanomeline–trospium (MD = −9.80; 95% CI: −13.00 to −6.60; *P* < .0001), and cariprazine (MD = −8.41; 95% CI, −11.33 to −5.50; *P* < .0001) showed statistically significant reductions in PANSS total score. Lumateperone, however, showed a nonsignificant trend toward better efficacy (MD = −2.79; 95% CI, −6.60 to 1.01; *P* = .15) (Figure 3). The heterogeneity analysis revealed moderate heterogeneity (*I*^2^ = 36.3%, tau^2^ = 3.19). The tests for heterogeneity within designs (*Q* = 12.57, df = 8, *P* = .13) and between designs (*Q* = 0.00, df = 0) were not significant, suggesting consistent treatment effects across studies. The transitivity assumption held, with a substantial baseline imbalance in age observed across treatment comparisons (*P* = .02). *P*-scores, which rank treatments based on their effectiveness, were highest for xanomeline–trospium (.8067), followed by asenapine (.8061), cariprazine (.63), and lumateperone (.24). Placebo consistently ranked lowest (Supplementary Material S8). This indicates xanomeline–trospium, asenapine, and cariprazine as the most effective treatments for acute exacerbation of schizophrenia among the interventions studied. The certainty of evidence was generally very low–low (Supplementary Material S9).

Sensitivity analysis was performed, including leave-one-out, excluding high risk of bias studies, and excluding small sample size studies, to assess the robustness of our network meta-analysis. Across all sensitivity models, xanomeline–trospium, asenapine, and cariprazine consistently showed the greatest efficacy in improving PANSS total scores. Importantly, heterogeneity decreased in multiple sensitivity analysis, with *I*^2^ dropped from 36.3% in the original results to 0% upon exclusion of Landbloom (2016), supporting the robustness of our data as this variability indicating a random error rather than a systematic differences across studies (Supplementary Material S10).

Moderate heterogeneity was observed for the primary outcome (*I*^2^ = 36.3%, tau^2^ = 3.19), suggesting some variability across studies. However, the network was globally consistent based on decomposition testing, with no statistically significant inconsistency detected.

### Safety Outcomes

#### Serious Adverse Events

**Figure 4:**
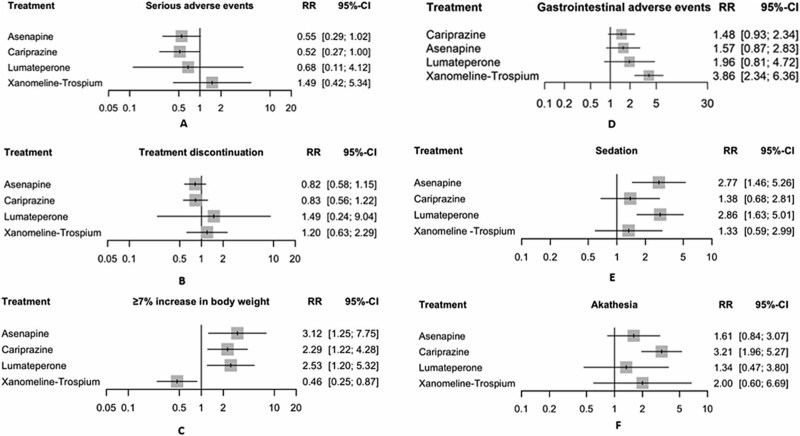
Safety outcomes. A series of forest plots compares the safety of cariprazine, asenapine, xanomeline–trospium chloride, and lumateperone treatments against placebo in patients with acute exacerbation of schizophrenia. The outcomes measured are (A) serious adverse events: The relative risk (RR) with 95% confidence intervals (CIs) shows the likelihood of experiencing serious adverse events for each treatment compared with placebo. (B) Treatment discontinuation: The RR with 95% CI indicates the likelihood of treatment discontinuation for each treatment compared with placebo. (C) ≥7% increase in body weight: The RR with 95% CI represents the likelihood of experiencing ≥ 7% increase in body weight for each treatment compared with placebo. (D) Gastrointestinal side effects: The RR with 95% CI shows the likelihood of experiencing gastrointestinal side effects for each treatment compared with placebo. (E) Sedation and/or somnolence: The RR with 95% CI shows the likelihood of experiencing sedation and/or somnolence for each treatment compared with placebo. (F) Akathisia: The RR with 95% CI shows the likelihood of experiencing akathisia for each treatment compared with placebo. Grey squares represent effect estimates; horizontal lines show 95% CIs

In the random effects model assessing serious adverse events (SAE), cariprazine showed a significant decrease in the risk of serious adverse events with a relative risk (RR) of 0.52 (95% CI, 0.2666-0.9974; *P* = .049) compared with placebo, indicating a better tolerability profile. Asenapine showed a nonsignificant trend toward reduced SAE risk, with a RR of 0.55 (95% CI, 0.2947-1.0163; *P* = .056). Interestingly, xanomeline–trospium showed a nonsignificant trend toward increased SAE risk with a RR of 1.49 (95% CI, 0.4181-5.3445; *P* = .546) (Figure 4A). The heterogeneity analysis revealed no significant heterogeneity, with *I*^2^ = 0%. Tests for heterogeneity within designs were not significant (*Q* = 6.82, df = 8, *P* = .556), indicating consistency among the study results. The transitivity assumption held, with a substantial baseline imbalance in age observed across treatment comparisons (*P* = .02). The *P*-scores, which rank treatments based on their effectiveness with higher scores indicating more favorable outcomes, were highest for cariprazine (.77), followed by asenapine (.73), lumateperone (.56), placebo (.28), and xanomeline–trospium (.17) (Supplementary  Material S8). The certainty of evidence was generally low (Supplementary Material S9).

**Table 2:** Baseline Characteristics of Patients

**Study ID**	**Age (mean, SD)**	**Male (%)**	**BMI (kg/m** ^**2**^ **) (mean, SD)**	**White (%)**	**PANSS total score (mean, SD)**	**CGI-S scale (mean, SD)**
Kinoshita (2016)^32^	41.42 ± 11.45	48.1	41.42 ± 11.45	NR	93.79 ± 17.51	NR
Landbloom (2016)^33^	40.6 ± 11.22	58.2	NR	72.7	94.2 ± 12.48	4.84 ± 0.60
Potkin (2007)^34^	40 (NR)	78.5	NR	39.2	94.45 (NR)	4.65 (NR)
Durgam (2014)^35^	36.42 ± 10.43	68.6	25.13 ± 4.54	51.4	97.08 ± 9.25	4.83 ± 1.18
Durgam (2015)^36^	38.23 ± 10.86	63.7	NR	63.7	96.1 ± 9.16	4.83 ± 0.62
Durgam (2016)^37^	41.3 ± 10.06	78.8	28.6 ± 5.65	32.4	95.0 ± 10.91	4.79 ± 0.68
Kane (2015)^38^	36.3 ± 10.4	76.5	24.2 ± 5.6	18.9	96.2 ± 9.2	4.9 ± 0.7
Correll (2020)^15^	42.4 ± 10.2	77.1	28.4 ± 5.3	26.1	89.8 ± 10.3	4.8 ± 0.6
Lieberman (2016)^16^	39.96 ± 9.69	80.2	NR	18.2	86.3 ± 11.98	NR
Brannan (2021)^41^	42.49 ± 10.15	76.9	28.86 ± 5.28	20.3	97.14 ± 9.05	4.95 ± 0.6
Kaul (2024_a)^39^	45.9 (10.6)	75	29.6 ± 5.4	23	98.1 ± 9.3	5.1 ± 0.6
Kaul (2024_b)^40^	43.1 ± 11.8	74.6	28.38 ± 5.4	38.3	97.0 ± 8.9	5.1 ± 0.6

Abbreviations: BMI, body mass index; CGI-S, Clinical Global Impressions-Severity of illness; PANSS, Positive and Negative Syndrome Scale; NR, Not Reported.

The table summarizes the baseline characteristics of patients included in the study. Characteristics include the number of participants, age, male participants, BMI, race, PANSS total score, and CGI-S scale. Mean values with SD are provided for continuous variables, while percentages are provided for categorical variables

#### Treatment Discontinuation due to Adverse Events

In the random effects model assessing treatment discontinuation due to adverse events, asenapine (RR = 0.82; 95% CI, 0.58-1.15; *P* = .25) and cariprazine (RR = 0.83; 95% CI, 0.56-1.22; *P* = .34) had a nonsignificant decrease in the risk of discontinuation compared with placebo. Lumateperone (RR = 1.49; 95% CI, 0.24-9.04; *P* = .67) and xanomeline–trospium (RR = 1.20; 95% CI, 0.63-2.29; *P* = .58) did not show a significant effect, although there was a trend toward increased risk (Figure 4B). The heterogeneity analysis revealed no significant heterogeneity, with *I*^2^ = 0%. Tests for heterogeneity within designs were not significant (*Q* = 6.49, df = 8, *P* = .59), indicating consistency among the study results. The transitivity assumption held, with a substantial baseline imbalance in age observed across treatment comparisons (*P* = .02). The *P*-scores, which rank treatments based on their effectiveness with higher scores indicating more favorable outcomes, were highest for asenapine (.74), followed by cariprazine (.72), placebo (.42), lumateperone (.32), and xanomeline–trospium (.30) (Supplementary  Material S8). The certainty of evidence was generally low–moderate (Supplementary Material S9).

#### ≥7% Increase in Body Weight

In the random effects model assessing the incidence of ≥ 7% increase in body weight, asenapine demonstrated a significant increase in the risk with a relative risk (RR) of 3.12 (95% CI, 1.25-7.75; *P* = .014), compared with placebo. Lumateperone also showed a significant increase in the risk of ≥ 7% increase in body weight with an RR of 2.53 (95% CI, 1.20-5.32; *P* = .014). This was followed by cariprazine with an RR 2.29 (95% CI, 1.22-4.28; *P* = .010). In contrast, xanomeline–trospium with an RR of 0.46 (95% CI, 0.25-0.87; *P* = .017) showed a significantly reduced risk (Figure 4C). The heterogeneity analysis revealed no significant heterogeneity, with *I*^2^ = 0%. Tests for heterogeneity within designs were not significant (*Q* = 3.43, df = 7, *P* = .84), indicating consistency among the study results. The transitivity assumption held, with a substantial baseline imbalance in age observed across treatment comparisons (*P* = .003). The *P*-scores, which rank treatments based on their effectiveness with higher scores indicating more favorable outcomes, were highest for xanomeline–trospium (.998), followed by placebo (.75), cariprazine (.32), lumateperone (.27), and asenapine (.51) (Supplementary Material S8). The certainty of evidence was generally moderate (Supplementary  Material S9).

#### Gastrointestinal Side Effects

In the random effects model assessing the incidence of gastrointestinal side effects including (nausea, vomiting, gastroesophageal reflux disease, abdominal pain, abdominal discomfort, oral hypoesthesia, dyspepsia, decreased appetite, dysgeusia, small intestine obstruction, diarrhea, and/or constipation), xanomeline–trospium showed the only significant increase in risk with a relative risk (RR) of 3.86 (95% CI, 2.34-6.36; *P* < .0001) compared with placebo. Asenapine (RR = 1.57; 95% CI, 0.87-2.83; *P* = .13), cariprazine (RR = 1.48; 95% CI, 0.93-2.34; *P* = .10), and lumateperone (RR = 1.96; 95% CI, 0.81-4.72; *P* = .14) all showed a nonsignificant increase in the risk of gastrointestinal side effects (Figure 4D). The heterogeneity analysis revealed substantial variability across studies, with an *I*^2^ of 67.20%. Tests of heterogeneity within designs were significant (*Q* = 24.4, df = 8, *P* = .002), indicating inconsistency among the study results. The transitivity assumption held, with a substantial baseline imbalance in age observed across treatment comparisons (*P* = .02). The *P*-scores, which rank treatments based on their effectiveness with higher scores indicating more favorable outcomes, were highest for placebo (.95), followed by cariprazine (.58), asenapine (.54), lumateperone (.40), and xanomeline–trospium (.03) (Supplementary Material S8).

#### Sedation and/or Somnolence

In the random effects model assessing the incidence of sedation and/or somnolence, lumateperone demonstrated a significant increase in the risk with a relative risk (RR) of 2.86 (95% CI, 1.63-5.01; *P* = .0003). Asenapine also showed a significant increase in the risk of sedation and/or somnolence with an RR of 2.77 (95% CI, 1.46-5.26; *P* = .0019). On the other hand, cariprazine (RR = 1.38; 95% CI, 0.68-2.81; *P* = .37) and xanomeline–trospium (RR = 1.33; 95% CI, 0.59-2.99; *P* = .49) did not show significant increase in the risk (Figure 4E). The heterogeneity analysis revealed moderate heterogeneity, with an *I*^2^ of 25.30%. Tests for heterogeneity within designs were not significant (*Q* = 8.03, df = 6, *P* = .24), indicating consistency among the study results. Sensitivity analyses using a leave-one-out analysis revealed that exclusion of either Kinoshita (2016) or Potkin (2007) reduced heterogeneity to 0%, suggesting that these were studies contributors to the observed heterogeneity. The transitivity assumption held, with no substantial baseline imbalances in age or sex observed across treatment comparisons. The *P*-scores, which rank treatments based on their effectiveness with higher scores indicating more favorable outcomes, were highest for placebo (.89), followed by xanomeline–trospium (.66), cariprazine (.63), asenapine (.17), and lumateperone (.15) (Supplementary Material S8). The certainty of evidence was generally low (Supplementary Material S9).

#### Akathisia

In the random effects model assessing the incidence of akathisia, cariprazine demonstrated a significant increase in the risk with a relative risk (RR) of 3.21 (95% CI, 1.96-5.27; *P* < .0001) compared with placebo. Asenapine (RR = 1.61; 95% CI, 0.84-3.07; *P* = .15), lumateperone (RR = 1.34; 95% CI, 0.47-3.80; *P* = .59), and xanomeline–trospium (RR = 2.00; 95% CI, 0.60-6.69; *P* = .26) did not show statistically significant differences (Figure 4F). The heterogeneity analysis revealed no significant heterogeneity, with *I*^2^ = 0%. Tests for heterogeneity within designs were not significant (*Q* = 6.28, df = 7, *P* = .51), indicating consistency among the study results. The transitivity assumption held, with a substantial baseline imbalance in age (*P* = .034) and sex (*P* = .008) observed across treatment comparisons. The *P*-scores, which rank treatments based on their effectiveness with higher scores indicating more favorable outcomes, were highest for placebo (.87), followed by lumateperone (.63), asenapine (.51), xanomeline–trospium (.40), and cariprazine (.09) (Supplementary Material S8). The certainty of evidence was generally low (Supplementary Material S9).

## Discussion

The findings of this network meta-analysis provide important insights into the efficacy and safety of asenapine, xanomeline–trospium, lumateperone, and cariprazine in the treatment of acute exacerbation of schizophrenia. Asenapine, xanomeline–trospium, and cariprazine showed effectiveness in terms of improvement in PANSS total score compared with placebo, with a pooled MD of −9.83 (95% CI, −13.49 to −6.18), −9.80 (95% CI, −13.00 to −6.60), and −8.41 (95% CI, −11.33 to −5.50), respectively. The differences between these 3 medications appear modest and unlikely to pose major clinical significance. In contrast, lumateperone did not show a statistically significant reduction in the PANSS total score. Asenapine and xanomeline–trospium ranked the highest overall among all agents analyzed, with nearly identical *P*-scores of .8061 and .8067, respectively (Supplementary Material S8). Although some treatments like asenapine, xanomeline–trospium, and cariprazine showed significant reductions in PANSS scores compared with placebo, the size of these changes may only reflect modest clinical benefit, depending on the total change from baseline. More research is needed to understand what level of placebo-adjusted improvement is truly meaningful for patients.

Safety analysis between asenapine and xanomeline–trospium, showed that asenapine had the most significant increase in body weight. Meanwhile, xanomeline–trospium was associated with an increased gastrointestinal side effect, a known side effect of this medication as previously reported in the literature.^14^ Notably, cariprazine was the only agent associated with a significantly reduced risk of serious adverse events, highlighting a potentially favorable safety profile in this regard.

To our knowledge, this study is the first to provide a practical comparison of asenapine, xanomeline–trospium, lumateperone, and cariprazine treatments for clinical practice, highlighting the significance of the efficacy and safety data. Our analysis provides a detailed assessment of PANSS total score. By providing information about adverse events, treatment discontinuation due to adverse events, and some highlights of the common adverse events, these findings underscore the importance of considering adverse event profiles and treatment discontinuation rates when selecting a therapeutic regimen.

Our findings are mostly consistent with previous pairwise meta-analyses that have reported beneficial effects of asenapine, xanomeline–trospium, and cariprazine treatments on PANSS total score reduction compared with placebo.^11^^,^^13^^,^^14^ While most studies in this network meta-analysis compared the included agents with placebo, some involving asenapine and cariprazine included active comparators such as olanzapine,^33^ risperidone,^34^^,^^35^ and airpiprazole.^36^ Prior comprehensive meta-analyses have consistently shown that both asenapine and cariprazine demonstrate similar efficacy to these standard agents, but not superior.^10^^,^^11^^,^^13^ While xanomeline–trospium has not been incorporated in direct head-to-head comparisons with other antipsychotics in any published meta-analysis. Taken together, these findings suggest asenapine and cariprazine are effective options for acute schizophrenia, but there is no strong evidence that they are more efficacious than traditional comparators. Clinicians should weigh efficacy alongside safety, tolerability, and individual patient factors in treatment selection.

In contrast to its statistically significant outcomes in individual RCTs,^15^^,^^16^ lumateperone, in our analysis, was not associated with a significant decrease in PANSS total score. Asenapine, in our analysis was found to be the highest in terms of weight gain, findings consistent with what has been previously reported and with its established antagonism at serotonergic receptors.^42^^,^^43^ However, a systemic review comparing it to other antipsychotics found that this increase in weight gain is relatively modest.^42^

This study has several notable strengths. We conducted a comprehensive literature search across major databases and included only RCTs, enhancing the internal validity of our findings. The use of a frequentist network meta-analysis allowed us to simultaneously compare multiple interventions and rank them based on efficacy. We applied robust methodological tools, including the CINeMA framework to evaluate the certainty of evidence and a structured transitivity assessment using baseline covariate balance.^31^ Our sensitivity analyses, including exclusion of high-risk studies, small sample size studies and leave-one-out tests, confirmed the stability of our conclusions (Supplementary Material S10).

Despite these strengths, our analysis has limitations. Key challenges include variability across studies in populations, interventions, and outcome measures, introducing heterogeneity and complicating direct comparisons. Although the overall risk-of-bias assessment was predominantly low across domains, a minority of studies provided insufficient detail on allocation concealment, or outcome assessor blinding, warranting cautious interpretation of their results (Supplementary Material S5). In addition, the network structure was anchored by placebo in most comparisons, and few studies directly compared active agents, limiting the strength of indirect comparisons. Publication bias could not be completely ruled out due to limited funnel plot asymmetry tests for small networks (Supplementary Material S6). Furthermore, reliance on published data may lead to publication bias, and the lack of individual patient data restricts detailed subgroup analyses. The short-term nature of the trial restricts the ability to draw conclusions regarding long-term efficacy and safety. Although negative symptoms are a key determinant of long-term functional outcomes in schizophrenia,^44-46^ we were unable to include them in our analysis due to lack of consistent reporting in the primary studies. Finally, our analysis focused only on asenapine, xanomeline–trospium, lumateperone, and cariprazine, excluding other antipsychotics that have been evaluated for acute schizophrenia, which may limit the comprehensiveness of our conclusion.

While our network meta-analysis identifies promising pharmacologic strategies for acute exacerbations of schizophrenia, several gaps remain. Studies with longer follow-up periods are also needed to assess the long-term tolerability and efficacy for treatments. Furthermore, 50% of patients with schizophrenia have comorbid psychiatric or medical conditions,^47^ the majority of trials excluded those patients, and also excluded those with substance use disorder, adolescents and young adults, those with predominant negative symptoms, treatment-refractory individuals, or those in stable phases of illness (Supplementary Material S3). Further trials that include these populations would enhance the generalizability and clinical applicability of future evidence. Finally, larger trials with expanded sample sizes and using validated scales are needed to guide clinical practice for this highly prevalent and damaging disorder.

## Conclusion

This network meta-analysis found that among asenapine, xanomeline–trospium, lumateperone and cariprazine, asenapine, xanomeline–trospium, and cariprazine were effective in reducing PANSS total score compared with placebo, whereas lumateperone did not show statistically significant reduction compared with placebo. These findings offer important insights into efficacy and safety of these 4 medications in the treatment of acute exacerbation of schizophrenia, though further head-to-head comparisons with the standard treatments are needed to guide a more evidence-based selection. Future trials with longer durations, and more diverse populations are warranted to confirm and extend these findings.

## Supplementary Material

Supplementary_Material_for_Review_sgaf024

## Data Availability

The data supporting the findings of this study are available from the corresponding author upon reasonable request.
