# Supplementary material for: Efficacy and Safety of Cariprazine, Asenapine, Xanomeline–Trospium, and Lumateperone for Acute Exacerbations of Schizophrenia in Adults: A Network Meta-Analysis
Source: Schizophr Bull Open. 2025 Oct 16;6(1):sgaf024. doi: 10.1093/schizbullopen/sgaf024 (PMC12617147; doi:10.1093/schizbullopen/sgaf024)
Supplement: Supplementary_Material_for_Review_sgaf024 [file supplementary_material_for_review_sgaf024.docx]

Table of Contents

Supplement S1. Search strategy4

Supplement S2. Network Plot of Treatment Comparisons 6

*Figure S2.1. Network Plot of Treatment Comparisons for PANSS total score6*

*Figure S2.2. Network Plot of Treatment Comparisons for treatment discontinuation7*

*Figure S2.3. Network Plot of Treatment Comparisons for serious adverse events8*

*Figure S2.4. Network Plot of Treatment Comparisons for ≥7 increase in body weight9*

*Figure S2.5. Network Plot of Treatment Comparisons for akathisia 10*

*Figure S2.6. Network Plot of Treatment Comparisons for sedation and/or somnolence11*

*Figure S2.7. Network Plot of Treatment Comparisons for gastrointestinal side effects12*

Supplement S3. Baseline characteristics of the included studies13

*Table S3. Study Characteristics and Outcomes in the included Clinical Trials13*

Supplement S4. Baseline Characteristics of the Participants19

*Table S4. Baseline Characteristics of Patients19*

Supplement S5. Risk of bias assessment of included trials20

*Table S5. Risk of bias assessment20*

Supplement S6. Publication bias (funnel plot)21

*Figure S6.1. PANSS total score21*

*Figure S6.2. Treatment discontinuation22*

*Figure S6.3. Serious adverse events23*

*Figure S6.4. ≥7 increase in body weight24*

*Figure S6.5. Akathisia25*

*Figure S6.6. Sedation and/or somnolence26*

*Figure S6.7. Gastrointestinal side effects27*

Supplement S7. League table for each outcome28

*Table S7.1. PANSS total score28*

*Table S7.2. Treatment discontinuation28*

*Table S7.3. Serious adverse events29*

*Table S7.4. ≥7 increase in body weight29*

*Table S7.5. Akathisia29*

*Table S7.6. Sedation and/or somnolence30*

*Table S7.7. Gastrointestinal side effects30*

Supplement S8. Treatment ranking for each outcome31

*Table S8.1. PANSS total score31*

*Table S8.2. Treatment discontinuation31*

*Table S8.3. Serious adverse events31*

*Table S8.4. ≥7 increase in body weight32*

*Table S8.5. Akathisia32*

*Table S8.6. Sedation and/or somnolence32*

*Table S8.7. Gastrointestinal side effects33*

Supplement S9. Certainty of evidence for each outcome34

Supplement S10. Sensitivity analysis43

*Table S10.1. Leave-One-Out Analysis for PNASS total score43*

*Table S10.2. Leave-One-Out Analysis for treatment discontinuation44*

*Table S10.3. Leave-One-Out Analysis for serious adverse events45*

*Table S10.4. Leave-One-Out Analysis for ≥7 increase in body weight46*

*Table S10.5. Leave-One-Out Analysis for akathisia47*

*Table S10.6. Leave-One-Out Analysis for sedation and/or somnolence48*

*Table S10.7. Leave-One-Out Analysis for gastrointestinal side effects49*

Supplement S11. PRISMA check list50

Supplement S1. Search strategy.

From inception February 8^th^, 2025

**- PubMed Search Syntax:**

(("schizophrenia" OR "psychosis" OR "acute psychosis" OR "schizophrenia exacerbation" OR "acute phase schizophrenia" OR "psychotic episode" OR "acute psychiatric decompensation")) AND (("Cariprazine" OR "Vraylar" OR "Asenapine" OR "Saphris" OR "Secuado" OR "Xanomeline-Trospium chloride" OR "KarXT" OR "Lumateperone" OR "Caplyta")) AND ("randomized controlled trial" OR RCT OR "clinical trial" OR "trial") AND ("PANSS" OR "PANSS positive subscale" OR "Simpson Angus" OR "adverse events" OR safety)

**- Scopus Search Syntax:**

(("schizophrenia" OR "psychosis" OR "acute psychosis" OR "schizophrenia exacerbation" OR "acute phase schizophrenia" OR "psychotic episode" OR "acute psychiatric decompensation")) AND (("Cariprazine" OR "Vraylar" OR "Asenapine" OR "Saphris" OR "Secuado" OR "Xanomeline-Trospium chloride" OR "KarXT" OR "Lumateperone" OR "Caplyta")) AND ("randomized controlled trial" OR RCT OR "clinical trial" OR "trial") AND ("PANSS" OR "PANSS positive subscale" OR "Simpson Angus" OR "adverse events" OR safety)

**- Cochrane Search Syntax:**

(("schizophrenia" OR "psychosis" OR "acute psychosis" OR "schizophrenia exacerbation" OR "acute phase schizophrenia" OR "psychotic episode" OR "acute psychiatric decompensation")) AND (("Cariprazine" OR "Vraylar" OR "Asenapine" OR "Saphris" OR "Secuado" OR "Xanomeline-Trospium chloride" OR "KarXT" OR "Lumateperone" OR "Caplyta")) AND ("randomized controlled trial" OR RCT OR "clinical trial" OR "trial") AND ("PANSS" OR "PANSS positive subscale" OR "Simpson Angus" OR "adverse events" OR safety)

**- Clinical Trials Search Syntax:**

(("schizophrenia" OR "psychosis" OR "acute psychosis" OR "schizophrenia exacerbation" OR "acute phase schizophrenia" OR "psychotic episode" OR "acute psychiatric decompensation")) AND (("Cariprazine" OR "Vraylar" OR "Asenapine" OR "Saphris" OR "Secuado" OR "Xanomeline-Trospium chloride" OR "KarXT" OR "Lumateperone" OR "Caplyta")) AND ("randomized controlled trial" OR RCT OR "clinical trial" OR "trial") AND ("PANSS" OR "PANSS positive subscale" OR "Simpson Angus" OR "adverse events" OR safety)

Supplement S2. Network plots of treatment comparisons

Figure S2.1. Network Plot of Treatment Comparisons for PANSS total score.

*
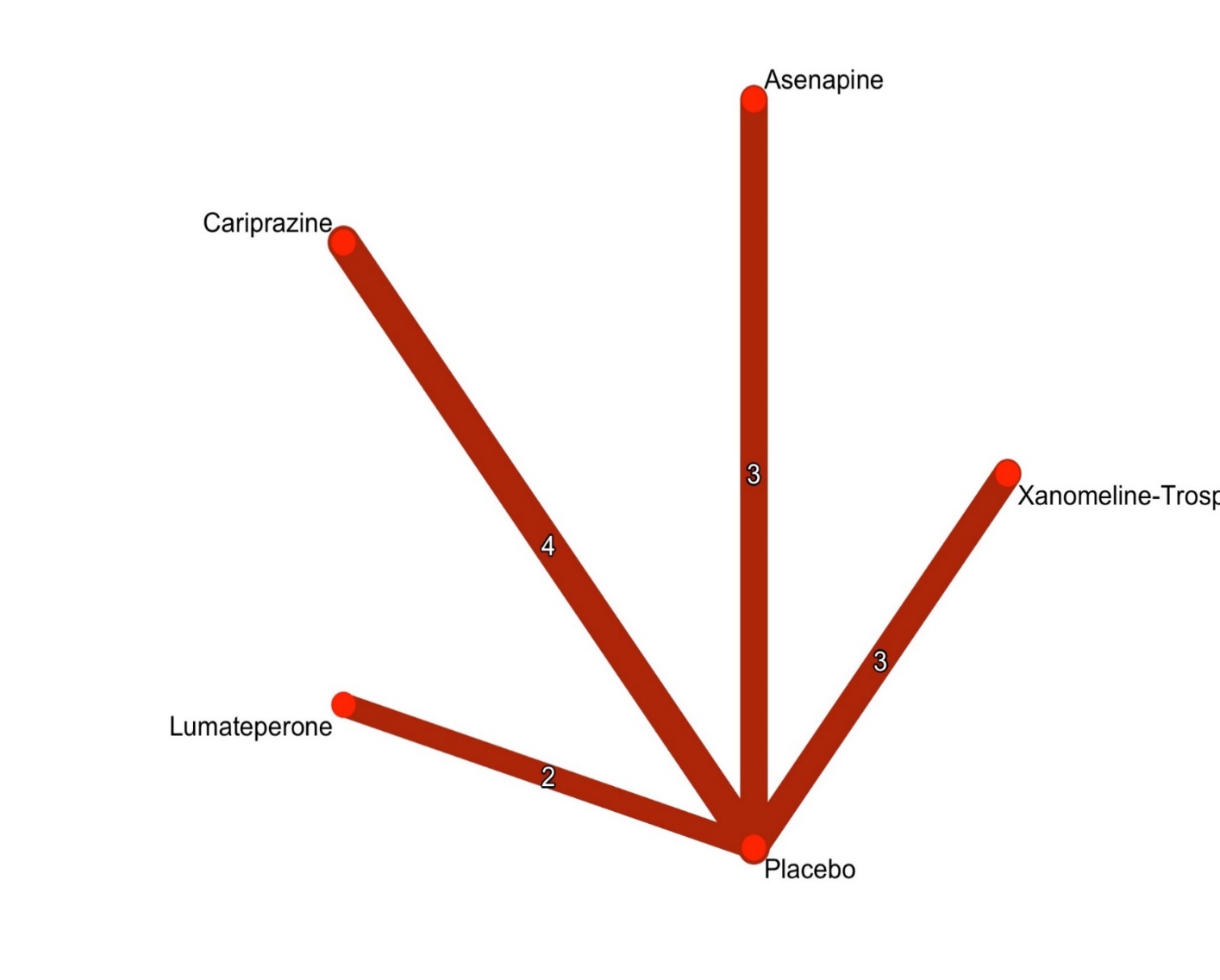
*

Figure S2.2. Network Plot of Treatment Comparisons for treatment discontinuation.


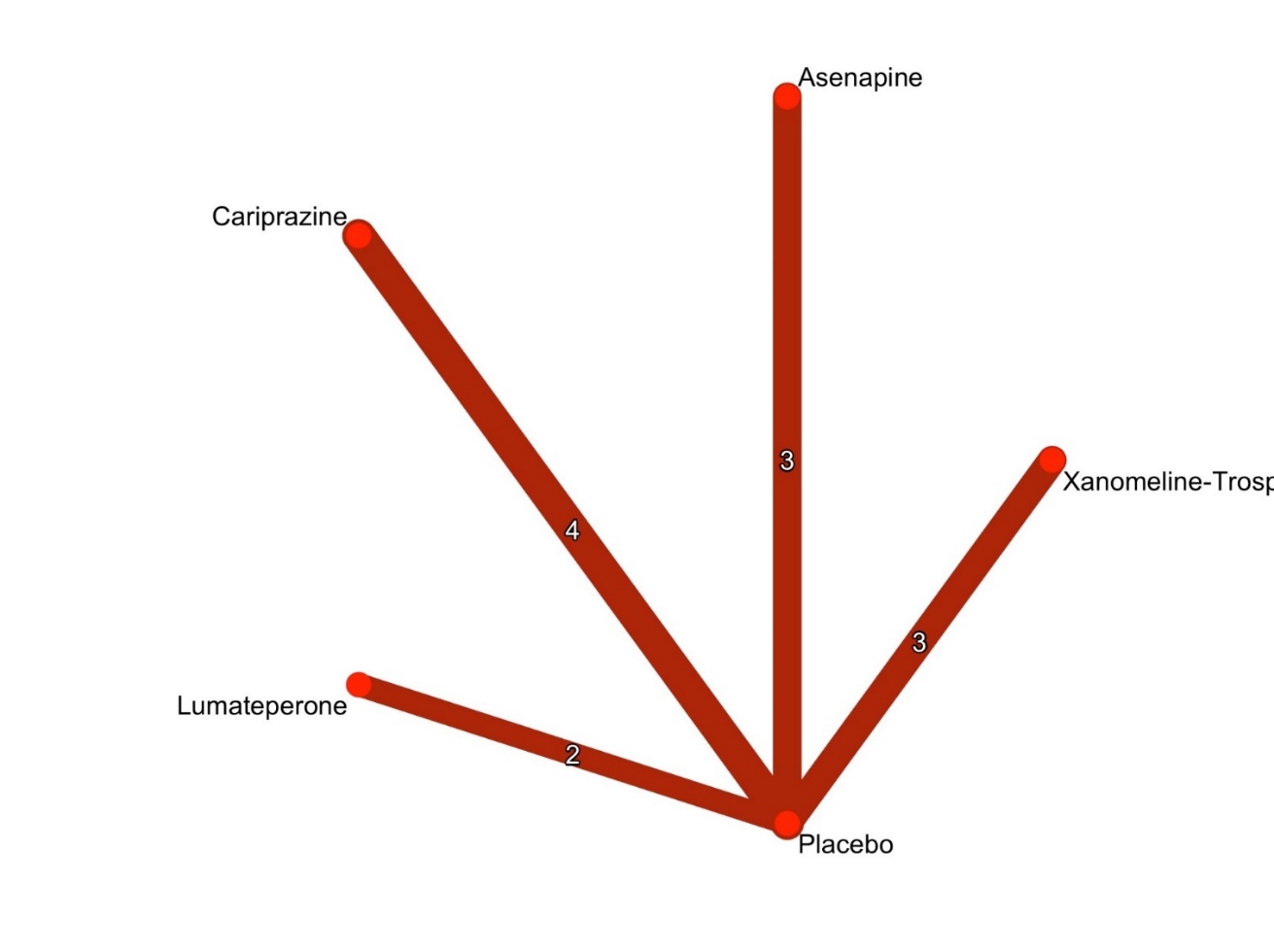


Figure S2.3. Network Plot of Treatment Comparisons for serious adverse events.


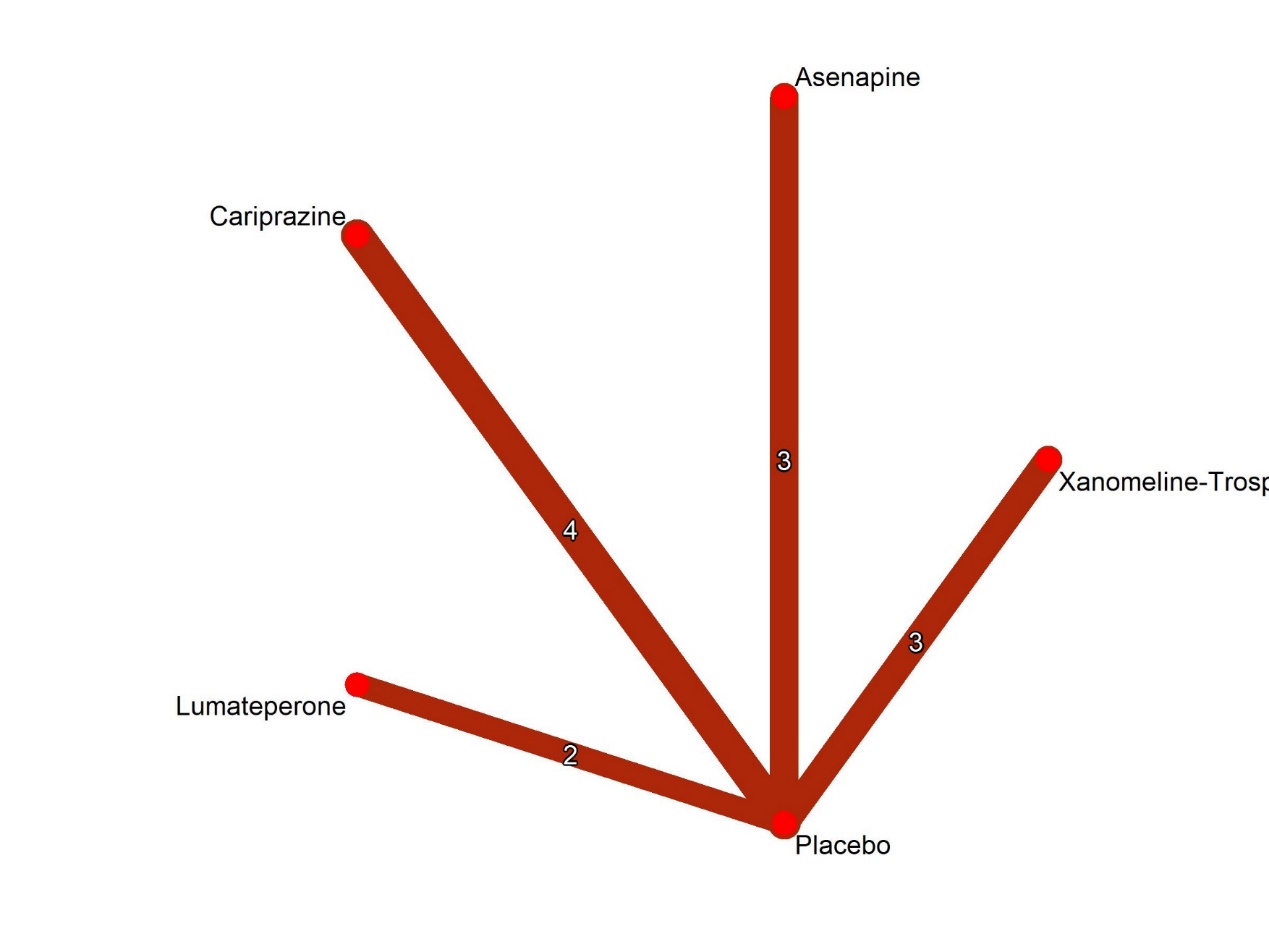


Figure S2.4. Network Plot of Treatment Comparisons for ≥7 increase in body weight.


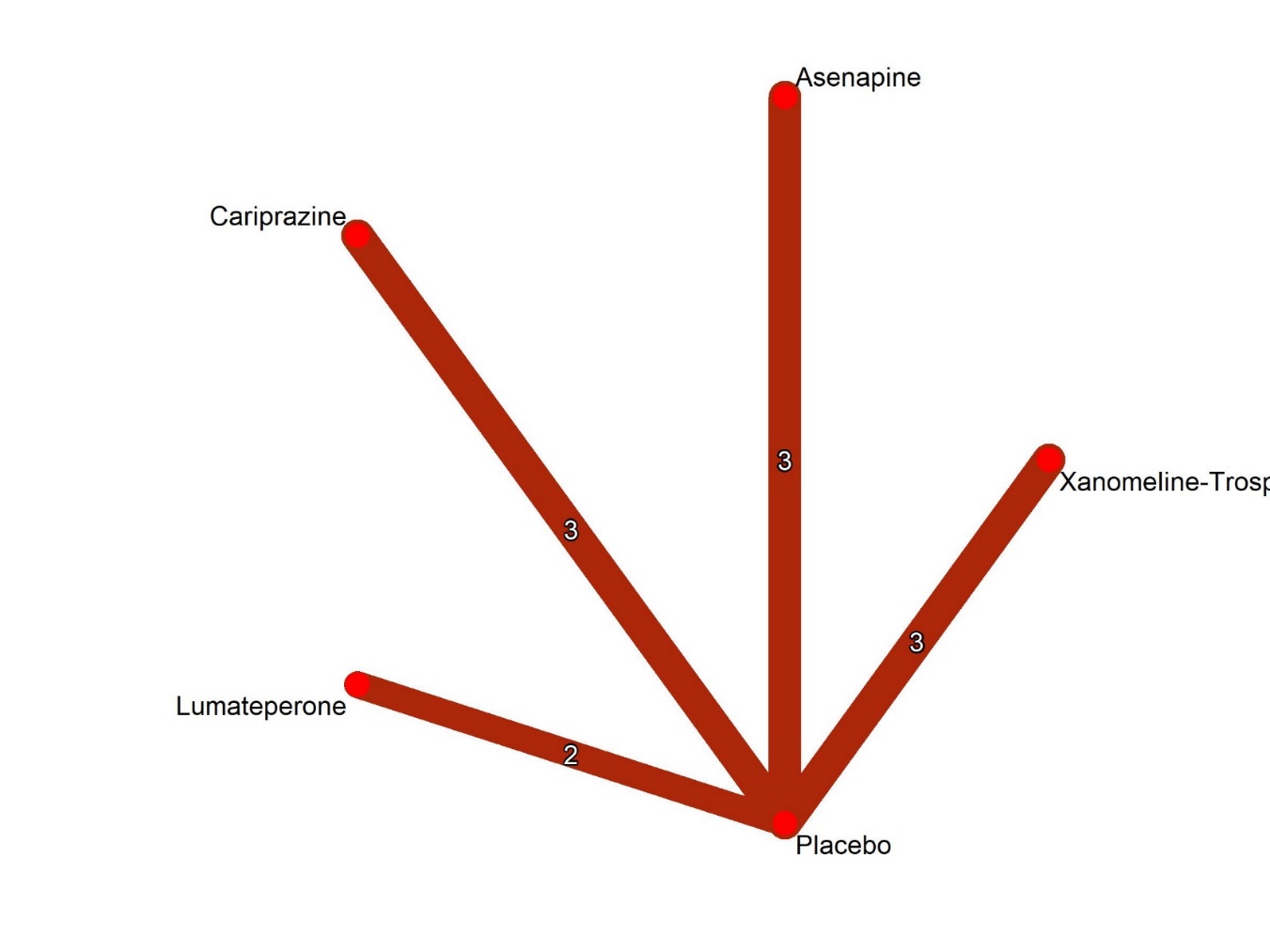


Figure S2.5. Network Plot of Treatment Comparisons for akathisia.


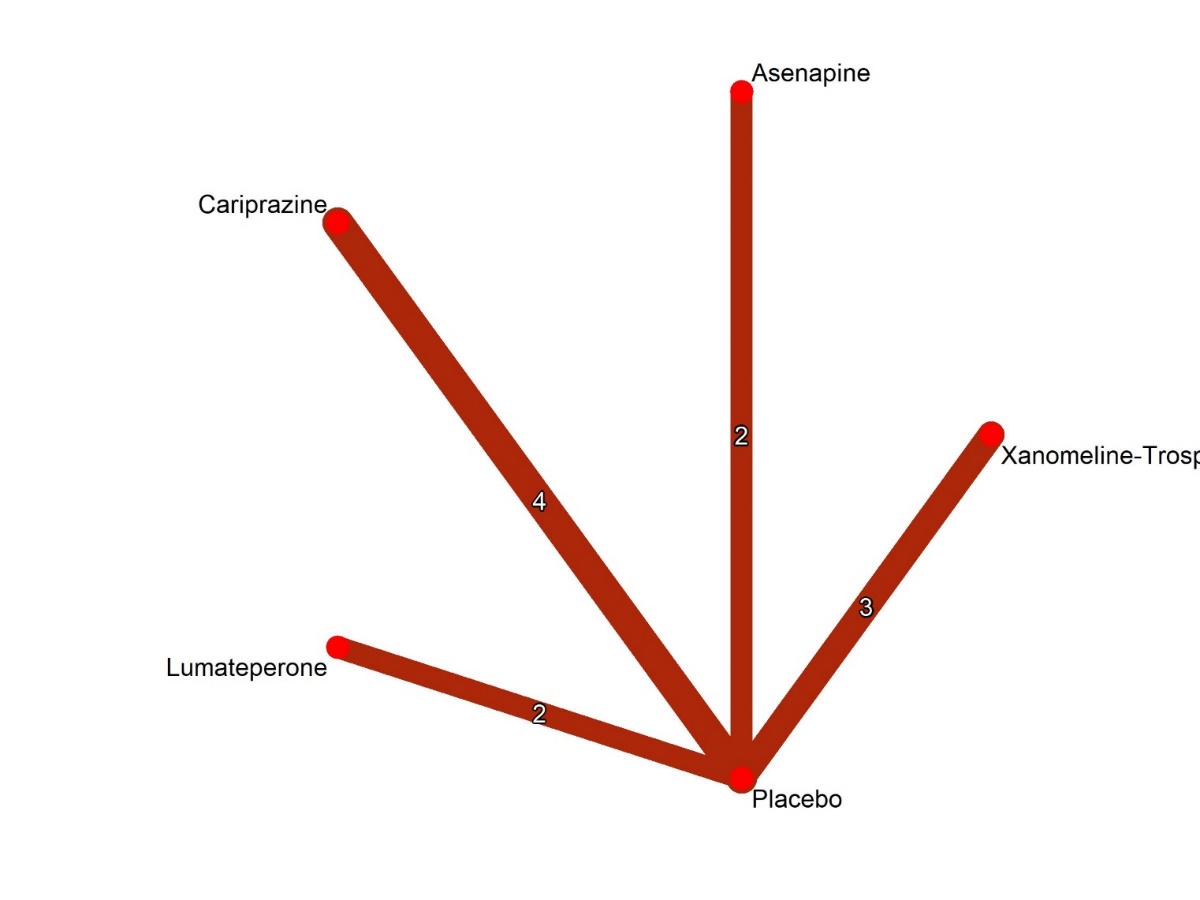


Figure S2.6. Network Plot of Treatment Comparisons for sedation and/or somnolence.


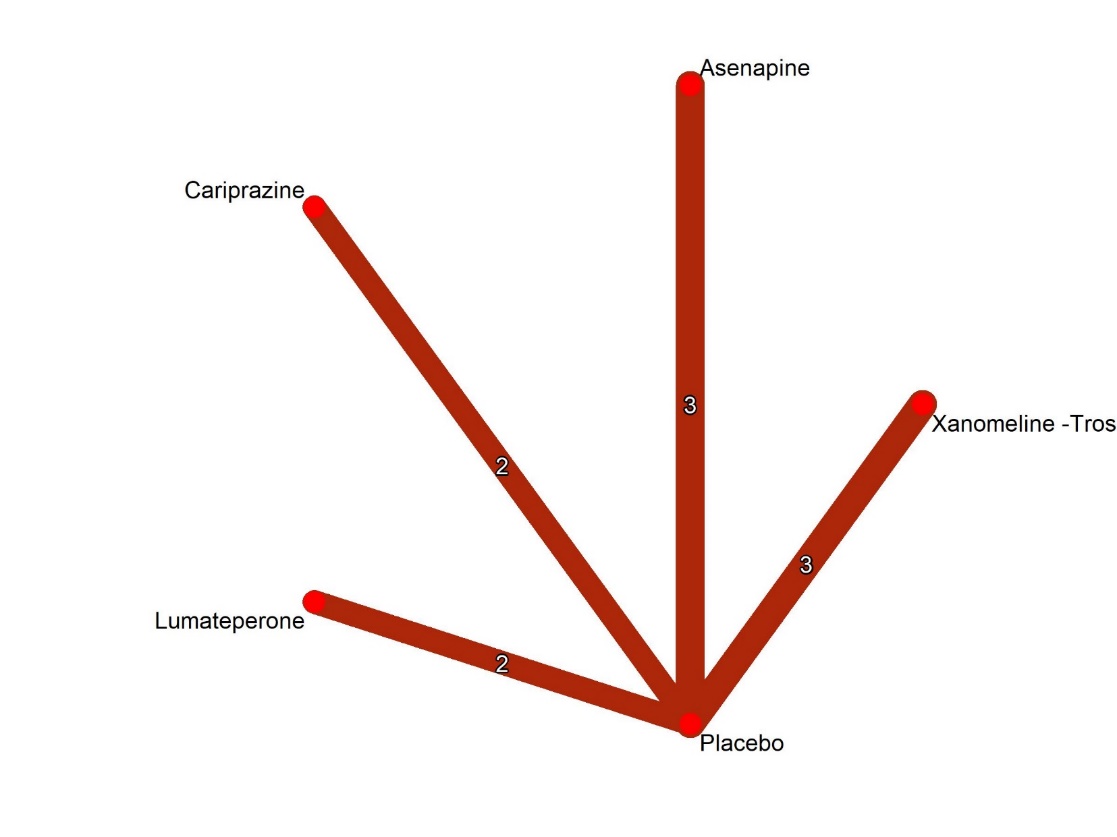


Figure S2.7. Network Plot of Treatment Comparisons for gastrointestinal side effects.


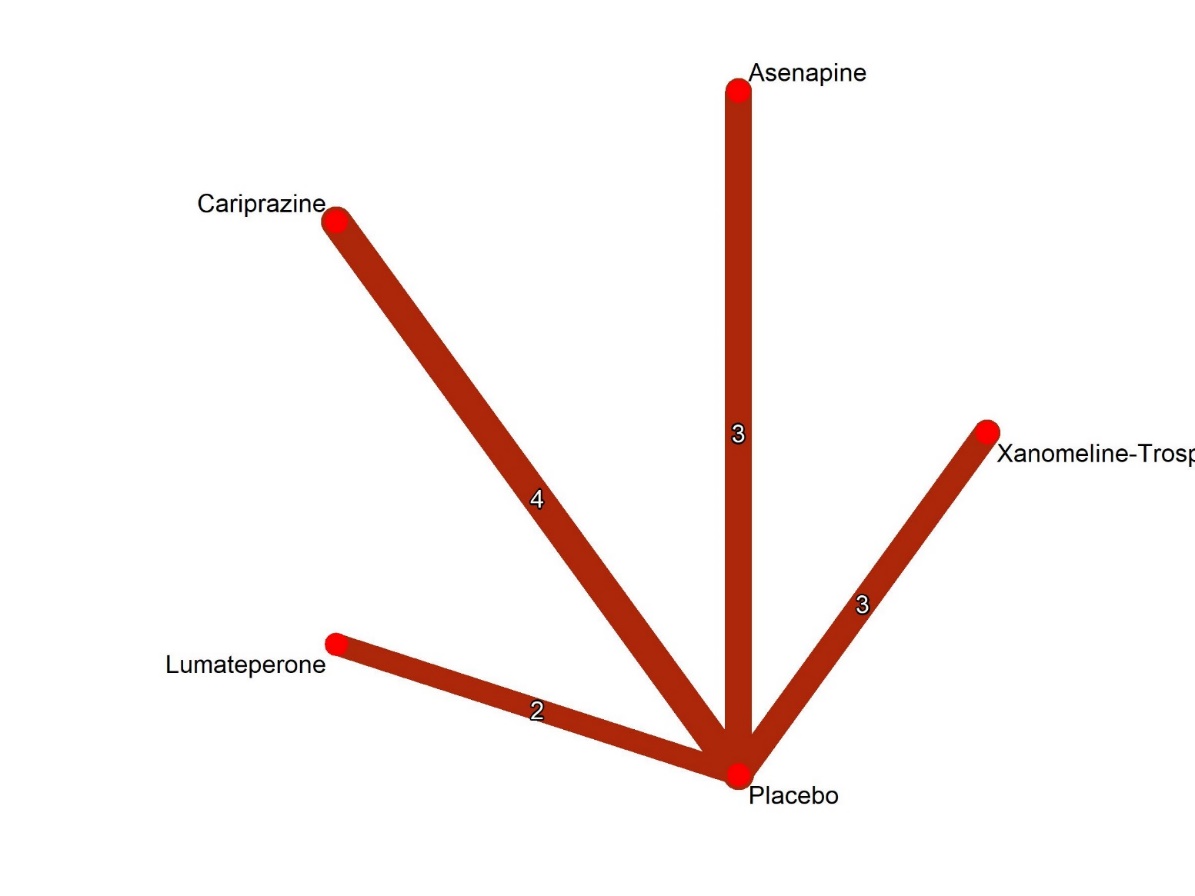


Supplement S3. Baseline characteristics of the included studies

Table S3: Study Characteristics and Outcomes in the included Clinical Trials

The table summarizes various clinical trials, detailing study design, registration, duration, treatment arms, primary and secondary outcomes, and population characteristics. Abbreviations used include PANSS (Positive and Negative Syndrome Scale), CGI-S (Clinical Global Impressions-Severity of illness), BPRS (Brief Psychiatric Rating Scale), and DSM (Diagnostic and Statistical Manual of Mental Disorders).

| **Study** | **Design** | **Registration** | **Duration** | **Treatment Arms** | **Primary Outcomes** | **Secondary Outcomes** | **Population Included** | **Population Excluded** |
| --- | --- | --- | --- | --- | --- | --- | --- | --- |
| Kinoshita,2016 | Phase III | NCT01098110 | 6 weeks | Asenapine (5 mg ,10 mg twice daily), Placebo | PANSS total score | PANSS subscale scores, CGI-S scale and responders, Safety | Asian patients 20 to 64 years with schizophrenia, exacerbation of ≤2 months, PANSS total score ≥60, CGI-S rating ≥4. | Any primary DSM-IV disorder other than schizophrenia. Patients with treatment-resistant schizophrenia (treatment with ≥3 antipsychotic drugs in the previous month), or the use of experimental medication within 12 weeks prior to baseline. Patients with a 20% or higher decrease in PANSS total score from screening to baseline, imminent risk of self-harm or harm to others, or previous participation in an asenapine trial were excluded. Typical treatment-related, concomitant medication, and medical/physical exclusions were applied. Substance abuse within the past 6 months was also an exclusion criterion. |
| Landbloom,2016 | Phase III | NCT01617187 | 6 weeks | Asenapine (2.5 mg ,5 mg twice daily), Olanzapine (15 mg once daily), Placebo | PANSS total score | CGI-S scale, Rate of PANSS responders, Safety | Patients (≥18 years) with schizophrenia, exacerbation of ≤8 weeks, PANSS total score ≥70, CGI-S rating ≥4. | Any primary DSM-IV disorder other than schizophrenia. Patients meeting DSM-IV-TR criteria for substance abuse/dependence (except nicotine) within the past 6 months. Patients with a >20% decrease in PANSS total score from screening to baseline, those at imminent risk of self-harm or harm to others. Patients who received prior treatment with asenapine for the current episode, were previously nonresponsive to asenapine, or were treated in a previous asenapine trial. Additionally, patients could not have taken clozapine within 12 weeks before baseline for treatment-resistant schizophrenia. Typical treatment-related, concomitant medication, and medical/physical exclusions were applied. |
| Potkin,2007 | NR | NR | 6 weeks | Asenapine (5 mg twice daily), Risperidone (3 mg twice daily), Placebo | PANSS total score | PANSS subscale scores, CGI-S scale, General psychopathology subscales, Safety | Patients (≥18 years) with schizophrenia, PANSS total score ≥60, CGI-S rating ≥4. | Any primary DSM-IV disorder other than schizophrenia. Patients with a history of treatment-resistant schizophrenia, or previous exposure to asenapine. use of experimental medication within 30 days before baseline. A score >2 (mild) on any item of AIMS at screening; or a diagnosis of drug and/or alcohol abuse (DSM-IV) within 30 days before screening. Typical treatment-related, concomitant medication, and medical/physical exclusions were applied. |
| Durgam,2014 | Phase II | NCT00694707 | 6 weeks | Cariprazine (1.5 mg daily), Cariprazine (3 mg daily), Cariprazine (4.5 mg daily), Risperidone (4 mg daily), Placebo | PANSS total score | PANSS subscale scores, CGI-S scale, Rate of PANSS responders, Safety | Patients 18 to 60 years with schizophrenia, exacerbation of ≤2 weeks, PANSS total score between 80 and 120, CGI-S rating ≥4. | Any primary DSM-IV disorder other than schizophrenia. Patients with treatment-resistant schizophrenia (poor response to ≥2 antipsychotics of adequate dose and duration) or suicidal or homicidal attempt/intent (active or preceding 2 years). alcohol or substance abuse or dependence within the past 3 months. Typical treatment-related, concomitant medication, and medical/physical exclusions were applied. |

Supplement S3. Cont.

| **Study** | **Design** | **Registration** | **Duration** | **Treatment Arms** | **Primary Outcomes** | **Secondary Outcomes** | **Population Included** | **Population Excluded** |
| --- | --- | --- | --- | --- | --- | --- | --- | --- |
| Durgam,2015 | Phase III | NCT01104766 | 6 weeks | Cariprazine (3 mg daily), Cariprazine (6 mg daily), Aripiprazole (10 mg daily), Placebo | PANSS total score | PANSS subscale scores, CGI-S scale, Rate of PANSS responders, Safety | Patients 18 to 60 years with schizophrenia, exacerbation of ≤2 weeks, PANSS total score between 80 and 120, CGI-S rating ≥4. | Any primary DSM-IV disorder other than schizophrenia. Patients with treatment-resistant schizophrenia (poor response to ≥2 antipsychotics of adequate dose and duration) or suicidal or homicidal attempt/intent (active or preceding 2 years). alcohol or substance abuse or dependence within the past 3 months. Typical treatment-related, concomitant medication, and medical/physical exclusions were applied. |
| Durgam,2016 | Phase II | NCT00404573 | 6 weeks | Cariprazine (1.5–4.5 mg daily), Cariprazine (6–12 mg daily), Placebo | PANSS total score | PANSS subscale scores, CGI-S scale, Safety | Patients 18 to 65 years with schizophrenia, exacerbation of ≤2 weeks, PANSS total score between 80 and 120, CGI-S rating ≥4. | Any primary DSM-IV disorder other than schizophrenia. Patients with treatment-resistant schizophrenia (poor response to ≥2 antipsychotics of adequate dose and duration) or suicidal or homicidal attempt/intent (active or preceding 2 years). alcohol or substance abuse or dependence within the past 3 months. Typical treatment-related, concomitant medication, and medical/physical exclusions were applied. |
| Kane,2015 | Phase III | NCT01104779 | 6 weeks | Cariprazine (3–6 mg daily), Cariprazine (6–9 mg daily), Placebo | PANSS total score | PANSS subscale scores, CGI-S scale, Safety | Patients 18 to 60 years with schizophrenia, exacerbation of ≤2 weeks, PANSS total score between 80 and 120, CGI-S rating ≥4. | Any primary DSM-IV disorder other than schizophrenia. Patients with treatment-resistant schizophrenia (poor response to ≥2 antipsychotics of adequate dose and duration) or suicidal or homicidal attempt/intent (active or preceding 2 years). alcohol or substance abuse or dependence within the past 3 months. Typical treatment-related, concomitant medication, and medical/physical exclusions were applied. |
| Correll,2020 | Phase III | NCT02282761 | 4 weeks | Lumateperone (28 mg daily), Lumateperone (42 mg daily), Placebo | PANSS total score | CGI-S scale, PANSS subscale scores, Personal and Social Performance, Safety | Patients 18 to 60 years with schizophrenia, exacerbation of ≤4 weeks, PANSS total score ≥70, CGI-S rating ≥4, BPRS total score ≥40. | Any primary DSM-5 disorder other than schizophrenia. Patients with substance abuse or dependence. Patients with imminent danger to self or others. Patients who received depot antipsychotics within 1.5 treatment cycles before baseline, any antipsychotic during the screening period, or specific agents with known interaction with 5-HT2A receptors. Patients with prior participation in a study with lumateperone or exposure to any investigational product within 3 months of Day −1. Typical treatment-related, concomitant medication, and medical/physical exclusions were applied. |

Supplement S3. Cont.

| **Study** | **Design** | **Registration** | **Duration** | **Treatment Arms** | **Primary Outcomes** | **Secondary Outcomes** | **Population Included** | **Population Excluded** |
| --- | --- | --- | --- | --- | --- | --- | --- | --- |
| Lieberman,2016 | Phase II | NCT01499563 | 4 weeks | Lumateperone (60 mg daily), Lumateperone (120 mg daily), Risperidone (4 mg daily), Placebo | PANSS total score | PANSS subscale scores, Rate of PANSS responders, Safety | Patients 18 to 60 years with schizophrenia, exacerbation of ≤4 weeks, BPRS total score ≥40. | Any primary DSM disorder other than schizophrenia. Patients with substance abuse or dependence, imminent danger to self or others. Patients who received depot antipsychotics within one treatment cycle before baseline, any antipsychotic during the screening period, or specific agents with known interaction with 5-HT2A receptors. Patients with prior participation in a study with ITI-007 or recent exposure to any investigational product. Typical treatment-related, concomitant medication, and medical/physical exclusions were applied. |
| Brannan,2021 | Phase II | NCT03697252 | 5 weeks | Xanomeline - Trospium (50-125 mg - 20-30 mg twice daily), Placebo | PANSS total score | PANSS subscale scores, CGI-S scale, Response according to CGI-S score, Safety | Patients 18 to 60 years with schizophrenia, exacerbation of ≤2 months, PANSS total score between 80 and 120, CGI-S score ≥4. | Patients diagnosed with any primary DSM-5 disorder other than schizophrenia within 12 months before screening. Patients with a DSM-5 diagnosis of moderate to severe substance use disorder within 12 months before screening or current abuse. Patients at risk for suicidal behavior. Patients with treatment-resistant schizophrenia. Typical treatment-related, concomitant medication, and medical/physical exclusions were applied. |
| Kaul,2024_a | Phase III | NCT04659161 | 5 weeks | Xanomeline - Trospium (50-125 mg - 20-30 mg twice daily), Placebo | PANSS total score | CGI- scale, PANSS subscale scores, Rate of PANSS responders, Safety | Patients 18 to 65 years with schizophrenia, exacerbation of ≤2 months, PANSS total score between 80 and 120, CGI-S score ≥4. | Patients diagnosed with any primary DSM-5 disorder other than schizophrenia within 12 months before screening were excluded. Patients with mild substance use disorder within 12 months before screening. Patients at risk for suicidal behavior. Patients currently receiving oral antipsychotics, or any other psychoactive medications. Patients with a history of treatment-resistant schizophrenia. Newly diagnosed or first-episode schizophrenia. Psychiatric hospitalization for more than 30 days (cumulative) within 90 days before screening; prior exposure to KarXT or adverse effects due to xanomeline or trospium; risk of violent or destructive behavior. Typical treatment-related, concomitant medication, and medical/physical exclusions were applied. |
| Kaul,2024_b | Phase III | NCT04738123 | 5 weeks | Xanomeline - Trospium (50-125 mg - 20-30 mg twice daily), Placebo | PANSS total score | CGI- scale, PANSS subscale scores, Rate of PANSS responders, Safety | Patients 18 to 65 years with schizophrenia, exacerbation of ≤2 months, PANSS total score between 80 and 120, CGI-S score ≥4. | Patients diagnosed with any primary DSM-5 disorder other than schizophrenia within 12 months before screening. Patients with mild substance use disorder within 12 months before screening. Patients at risk for suicidal behavior. Patients currently receiving oral antipsychotics, or other psychoactive medications. Participation in another clinical study within 3 months of screening. Patients with treatment-resistant schizophrenia (failure to respond to 2 adequate pharmacotherapy courses or requiring clozapine within the last 12 months). Typical treatment-related, concomitant medication, and medical/physical exclusions were applied. |

Supplement S4. Baseline Characteristics of the Participants

Table S4. Baseline Characteristics of Patients

The table summarizes the baseline characteristics of patients included in the study. Characteristics include the number of participants, age, male participants, body mass index (BMI), race, Positive and Negative Syndrome Scale (PANSS total score) , and Clinical Global Impressions-Severity of illness (CGI-S scale). Mean values with SD are provided for continuous variables, while percentages are provided for categorical variables. Abbreviations: BMI: Body mass index; CGI-S (Clinical Global Impressions-Severity of illness); PANSS (Positive and Negative Syndrome Scale).

| **Study ID** | **Participants** | **Age (mean, SD)** | **Male (%)** | **BMI (kg/m²) (mean, SD)** | **White (%)** | **PANSS total score (mean, SD)** | **CGI-S scale (mean, SD)** |
| --- | --- | --- | --- | --- | --- | --- | --- |
| Kinoshita,2016 | 532 | 41.42 ± 11.45 | 48.1% | 41.42 ± 11.45 | NR | 93.79 ± 17.51 | NR |
| Landbloom,2016 | 360 | 40.6 ± 11.22 | 58.2% | NR | 72.7% | 94.2 ± 12.48 | 4.84 ± 0.60 |
| Potkin,2007 | 182 | 40 (NR) | 78.5% | NR | 39.2% | 94.45 (NR) | 4.65 (NR) |
| Durgam,2014 | 732 | 36.42 ± 10.43 | 68.6% | 25.13 ± 4.54 | 51.4% | 97.08 ± 9.25 | 4.83 ± 1.18 |
| Durgam,2015 | 465 | 38.23 ± 10.86 | 63.7% | NR | 63.7% | 96.1 ± 9.16 | 4.83 ± 0.62 |
| Durgam,2016 | 392 | 41.3 ± 10.06 | 78.8% | 28.6 ± 5.65 | 32.4% | 95.0 ± 10.91 | 4.79 ± 0.68 |
| Kane,2015 | 446 | 36.3 ± 10.4 | 76.5% | 24.2 ± 5.6 | 18.9% | 96.2 ± 9.2 | 4.9 ± 0.7 |
| Correll,2020 | 450 | 42.4 ± 10.2 | 77.1% | 28.4 ± 5.3 | 26.1% | 89.8 ± 10.3 | 4.8 ± 0.6 |
| Lieberman,2016 | 335 | 39.96 ± 9.69 | 80.2% | NR | 18.2% | 86.3 ± 11.98 | NR |
| Brannan,2021 | 182 | 42.49 ± 10.15 | 76.9% | 28.86 ± 5.28 | 20.3% | 97.14 ± 9.05 | 4.95 ± 0.6 |
| Kaul,2024_a | 252 | 45.9 (10.6) | 75% | 29.6 ± 5.4 | 23% | 98.1 ± 9.3 | 5.1 ± 0.6 |
| Kaul,2024_b | 256 | 43.1 ± 11.8 | 74.6% | 28.38 ± 5.4 | 38.3% | 97.0 ± 8.9 | 5.1 ± 0.6 |

Supplement S5. Risk of bias assessment of included trials.

Table S5: Risk of bias assessment

| **Study ID** | **Random Sequence Generation** | **Allocation Concealment** | **Blinding of Participants/Personnel** | **Blinding of Outcome Assessment** | **Incomplete Outcome Data** | **Selective Reporting** | **Other Bias** | **Overall Judgment** |
| --- | --- | --- | --- | --- | --- | --- | --- | --- |
| Kinoshita,2016 | Low Risk | Some Concerns | Low Risk | Some Concerns | Some Concerns | Low Risk | Some Concerns | Some Concerns |
| Landbloom,2016 | Low Risk | Low Risk | Low Risk | Some Concerns | Some Concerns | Low Risk | Some Concerns | Some Concerns |
| Potkin,2007 | Some Concerns | Some Concerns | Low Risk | Some Concerns | Low Risk | Some Concerns | Some Concerns | Some Concerns |
| Durgam,2014 | Some Concerns | Some Concerns | Low Risk | Some Concerns | Low Risk | Low Risk | Some Concerns | Some Concerns |
| Durgam,2015 | Some Concerns | Some Concerns | Low Risk | Low Risk | Low Risk | Low Risk | Some Concerns | Some Concerns |
| Durgam,2016 | Some Concerns | Some Concerns | Low Risk | Some Concerns | Low Risk | Low Risk | Some Concerns | Some Concerns |
| Kane,2015 | Some Concerns | Some Concerns | Low Risk | Low Risk | Low Risk | Low Risk | Some Concerns | Some Concerns |
| Correll,2020 | Low Risk | Low Risk | Low Risk | Low Risk | Low Risk | Low Risk | Some Concerns | Some Concerns |
| Lieberman,2016 | Low Risk | Low Risk | Low Risk | Low Risk | Low Risk | Low Risk | Some Concerns | Some Concerns |
| Brannan,2021 | Low Risk | Low Risk | Low Risk | Low Risk | Low Risk | Low Risk | Some Concerns | Some Concerns |
| Kaul,2024_a | Low Risk | Low Risk | Low Risk | Low Risk | Low Risk | Low Risk | Some Concerns | Some Concerns |
| Kaul,2024_b | Low Risk | Low risk | Low Risk | Low Risk | Low Risk | High Risk | Some Concerns | High Risk |

Supplement S6. Publication bias (funnel plot)

Figure S6.1. PANSS total score.


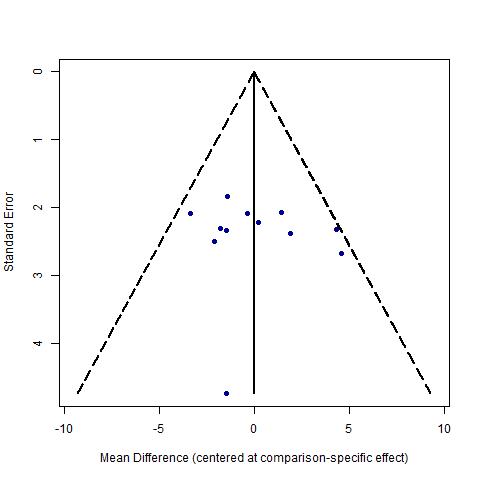


Figure S6.2. Treatment discontinuation.


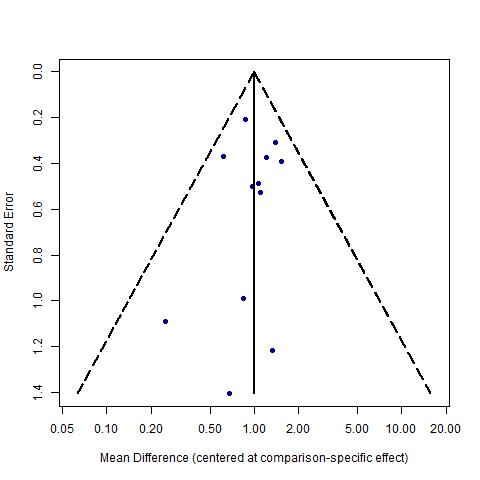


Figure S6.3. Serious adverse events.


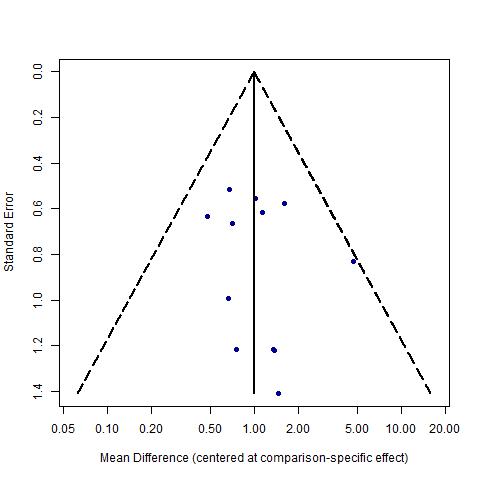


Figure S6.4. ≥7 increase in body weight.


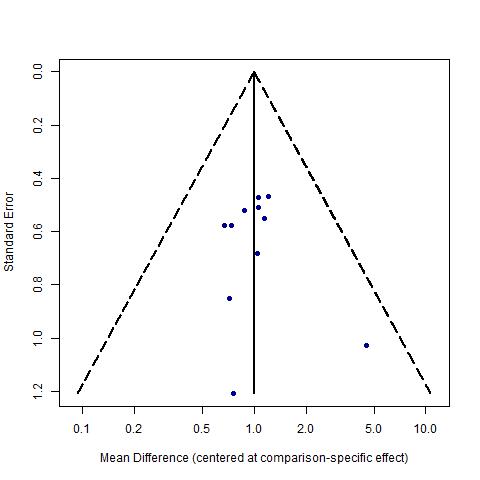


Figure S6.5. Akathisia


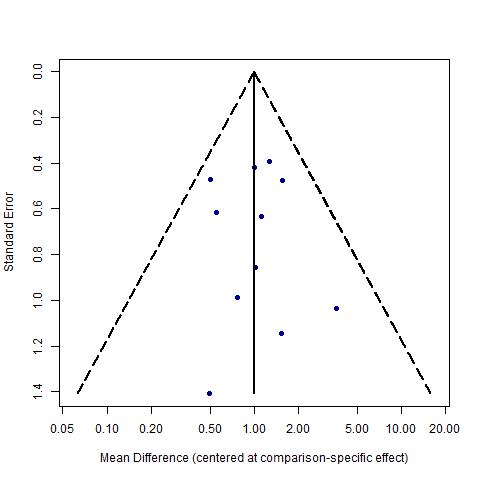


Figure S6.6. Sedation and/or somnolence.


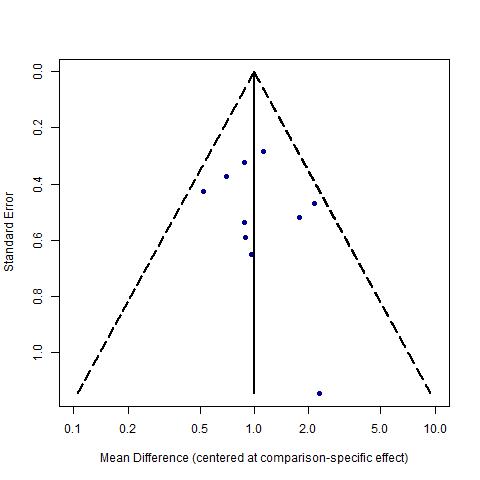


Figure S6.7. Gastrointestinal side effects.


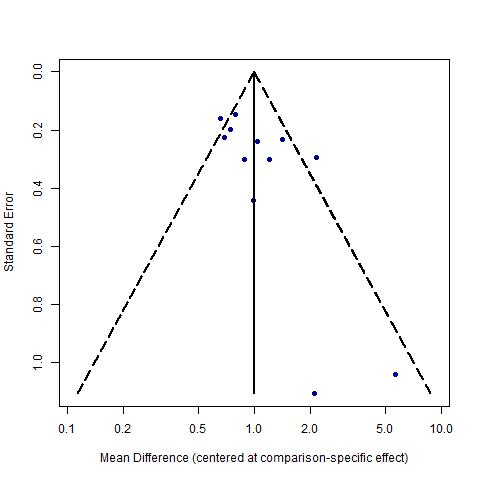


Supplement S7. League table for each outcome.

Table S7.1. for PANSS total score.

|  | V1 | V2 | V3 | V4 | V5 |
| --- | --- | --- | --- | --- | --- |
| 1 | Asenapine | . | . | . | -9.91 (-12.80; -7.02) |
| 2 | -0.15 ( -3.96; 3.66) | Xanomeline-Trospium | . | . | -9.76 (-12.24; -7.28) |
| 3 | -1.42 ( -5.13; 2.29) | -1.27 ( -4.66; 2.13) | Cariprazine | . | -8.49 (-10.81; -6.17) |
| 4 | -6.95 (-11.01; -2.88) | -6.79 (-10.57; -3.01) | -5.52 ( -9.20; -1.84) | Lumateperone | -2.97 ( -5.82; -0.11) |
| 5 | -9.91 (-12.80; -7.02) | -9.76 (-12.24; -7.28) | -8.49 (-10.81; -6.17) | -2.97 ( -5.82; -0.11) | Placebo |

Table S7.2. for treatment discontinuation.

|  | V1 | V2 | V3 | V4 | V5 |
| --- | --- | --- | --- | --- | --- |
| 1 | Asenapine | . | 0.82 (0.58; 1.15) | . | . |
| 2 | 0.99 (0.59; 1.66) | Cariprazine | 0.83 (0.56; 1.22) | . | . |
| 3 | 0.82 (0.58; 1.15) | 0.83 (0.56; 1.22) | Placebo | 0.67 (0.11; 4.09) | 0.83 (0.44; 1.59) |
| 4 | 0.55 (0.09; 3.46) | 0.56 (0.09; 3.53) | 0.67 (0.11; 4.09) | Lumateperone | . |
| 5 | 0.68 (0.33; 1.42) | 0.69 (0.33; 1.47) | 0.83 (0.44; 1.59) | 1.24 (0.18; 8.45) | Xanomeline-Trospium |

Table S7.3. for serious adverse events.

|  | V1 | V2 | V3 | V4 | V5 |
| --- | --- | --- | --- | --- | --- |
| 1 | Cariprazine | . | . | 0.52 (0.27; 1.00) | . |
| 2 | 0.94 (0.38; 2.33) | Asenapine | . | 0.55 (0.29; 1.02) | . |
| 3 | 0.76 (0.11; 5.18) | 0.81 (0.12; 5.42) | Lumateperone | 0.68 (0.11; 4.12) | . |
| 4 | 0.52 (0.27; 1.00) | 0.55 (0.29; 1.02) | 0.68 (0.11; 4.12) | Placebo | 0.67 (0.19; 2.39) |
| 5 | 0.34 (0.08; 1.45) | 0.37 (0.09; 1.51) | 0.45 (0.05; 4.13) | 0.67 (0.19; 2.39) | Xanomeline-Trospium |

Table S7.4. for ≥7 increase in body weight.

|  | V1 | V2 | V3 | V4 | V5 |
| --- | --- | --- | --- | --- | --- |
| 1 | Xanomeline-Trospium | 0.46 (0.25; 0.87) | . | . | . |
| 2 | 0.46 (0.25; 0.87) | Placebo | 0.44 (0.23; 0.82) | 0.40 (0.19; 0.83) | 0.32 (0.13; 0.80) |
| 3 | 0.20 (0.08; 0.49) | 0.44 (0.23; 0.82) | Cariprazine | . | . |
| 4 | 0.18 (0.07; 0.49) | 0.40 (0.19; 0.83) | 0.90 (0.34; 2.39) | Lumateperone | . |
| 5 | 0.15 (0.05; 0.45) | 0.32 (0.13; 0.80) | 0.73 (0.24; 2.22) | 0.81 (0.25; 2.63) | Asenapine |

Table S7.5. for akathisia.

|  | V1 | V2 | V3 | V4 | V5 |
| --- | --- | --- | --- | --- | --- |
| 1 | Placebo | 0.75 (0.26; 2.13) | 0.62 (0.33; 1.19) | 0.50 (0.15; 1.68) | 0.31 (0.19; 0.51) |
| 2 | 0.75 (0.26; 2.13) | Lumateperone | . | . | . |
| 3 | 0.62 (0.33; 1.19) | 0.83 (0.24; 2.85) | Asenapine | . | . |
| 4 | 0.50 (0.15; 1.68) | 0.67 (0.14; 3.31) | 0.80 (0.20; 3.17) | Xanomeline-Trospium | . |
| 5 | 0.31 (0.19; 0.51) | 0.42 (0.13; 1.32) | 0.50 (0.22; 1.13) | 0.62 (0.17; 2.30) | Cariprazine |

Table S7.6. for sedation and/or somnolence.

|  | V1 | V2 | V3 | V4 | V5 |
| --- | --- | --- | --- | --- | --- |
| 1 | Placebo | 0.76 (0.37; 1.59) | 0.75 (0.42; 1.36) | 0.37 (0.21; 0.64) | 0.35 (0.23; 0.53) |
| 2 | 0.76 (0.37; 1.59) | Xanomeline -Trospium | . | . | . |
| 3 | 0.75 (0.42; 1.36) | 0.99 (0.38; 2.54) | Cariprazine | . | . |
| 4 | 0.37 (0.21; 0.64) | 0.48 (0.19; 1.21) | 0.49 (0.22; 1.10) | Asenapine | . |
| 5 | 0.35 (0.23; 0.53) | 0.46 (0.20; 1.06) | 0.46 (0.22; 0.96) | 0.95 (0.47; 1.91) | Lumateperone |

Table S7.7. for gastrointestinal side effects.

|  | V1 | V2 | V3 | V4 | V5 |
| --- | --- | --- | --- | --- | --- |
| 1 | Placebo | 0.76 (0.60; 0.95) | 0.72 (0.53; 0.96) | 0.55 (0.31; 0.97) | 0.29 (0.23; 0.36) |
| 2 | 0.76 (0.60; 0.95) | Cariprazine | . | . | . |
| 3 | 0.72 (0.53; 0.96) | 0.95 (0.65; 1.38) | Asenapine | . | . |
| 4 | 0.55 (0.31; 0.97) | 0.72 (0.39; 1.34) | 0.76 (0.40; 1.46) | Lumateperone | . |
| 5 | 0.29 (0.23; 0.36) | 0.39 (0.28; 0.53) | 0.41 (0.28; 0.59) | 0.53 (0.29; 0.99) | Xanomeline-Trospium |

Supplement S8. Treatment ranking for each outcome.

Table S8.1. for PANSS total score.

| **Rank** | **Intervention** | **P-score** |
| --- | --- | --- |
| First | Xanomeline-Trospium | 0.8067 |
| Second | Asenapine | 0.8061 |
| Third | Cariprazine | 0.6326 |
| Fourth | Lumateperone | 0.2357 |
| Fifth | Placebo | 0.0188 |

Table S8.2. for treatment discontinuation.

| **Rank** | **Intervention** | **P-score** |
| --- | --- | --- |
| First | Asenapine | 0.7441 |
| Second | Cariprazine | 0.7197 |
| Third | Placebo | 0.4170 |
| Fourth | Lumateperone | 0.3187 |
| Fifth | Xanomeline-Trospium | 0.3005 |

Table S8.3. for serious adverse events.

| **Rank** | **Intervention** | **P-score** |
| --- | --- | --- |
| First | Cariprazine | 0.7661 |
| Second | Asenapine | 0.7316 |
| Third | Lumateperone | 0.5556 |
| Fourth | Placebo | 0.2804 |
| Fifth | Xanomeline-Trospium | 0.1663 |

Table S8.4. for ≥7 increase in body weight.

| **Rank** | **Intervention** | **P-score** |
| --- | --- | --- |
| First | Xanomeline-Trospium | 0.9977 |
| Second | Placebo | 0.7473 |
| Third | Cariprazine | 0.3235 |
| Fourth | Lumateperone | 0.2659 |
| Fifth | Asenapine | 0.1657 |

Table S8.5. for akathisia.

| **Rank** | **Intervention** | **P-score** |
| --- | --- | --- |
| First | Placebo | 0.8748 |
| Second | Lumateperone | 0.6325 |
| Third | Asenapine | 0.5087 |
| Fourth | Xanomeline-Trospium | 0.3952 |
| Fifth | Cariprazine | 0.0888 |

Table S8.6. for sedation and/or somnolence.

| **Rank** | **Intervention** | **P-score** |
| --- | --- | --- |
| First | Placebo | 0.8927 |
| Second | Xanomeline -Trospium | 0.6563 |
| Third | Cariprazine | 0.6299 |
| Fourth | Asenapine | 0.1725 |
| Fifth | Lumateperone | 0.1486 |

Table S8.7. for gastrointestinal side effects.

| **Rank** | **Intervention** | **P-score** |
| --- | --- | --- |
| First | Placebo | 0.9546 |
| Second | Cariprazine | 0.5802 |
| Third | Asenapine | 0.5363 |
| Fourth | Lumateperone | 0.4018 |
| Fifth | Xanomeline-Trospium | 0.0270 |

Supplement S9. Certainty of evidence for each outcome.

Table S9.1 for PANSS total score

| **Comparison** | **Within Study Bias** | **Reporting Bias** | **Indirectness** | **Imprecision** | **Heterogeneity** | **Incoherence** | **Overall Confidence** | **Reason for downgrade** |
| --- | --- | --- | --- | --- | --- | --- | --- | --- |
| Asenapine:Placebo | Some concerns | Some concerns | No concerns | Some concerns | Major concerns | No concerns | Low | Within-study bias, Reporting bias, Imprecision, Heterogeneity |
| Cariprazine:Placebo | Some concerns | Some concerns | No concerns | Some concerns | Major concerns | No concerns | Low | Within-study bias, Reporting bias, Imprecision, Heterogeneity |
| Lumateperone:Placebo | Some concerns | Some concerns | No concerns | Major concerns | Major concerns | No concerns | Very low | Within-study bias, Reporting bias, Imprecision, Heterogeneity |
| Placebo:Xanomeline-Trospium | Some concerns | Some concerns | No concerns | Some concerns | Major concerns | No concerns | Low | Within-study bias, Reporting bias, Imprecision, Heterogeneity |
| Asenapine:Cariprazine | Some concerns | Some concerns | No concerns | Major concerns | Major concerns | No concerns | Very low | Within-study bias, Reporting bias, Imprecision, Heterogeneity |
| Asenapine:Lumateperone | Some concerns | Some concerns | No concerns | Some concerns | Major concerns | No concerns | Low | Within-study bias, Reporting bias, Imprecision, Heterogeneity |
| Asenapine:Xanomeline-Trospium | Some concerns | Some concerns | No concerns | Major concerns | Major concerns | No concerns | Very low | Within-study bias, Reporting bias, Imprecision, Heterogeneity |
| Cariprazine:Lumateperone | Some concerns | Some concerns | No concerns | Some concerns | Major concerns | No concerns | Low | Within-study bias, Reporting bias, Imprecision, Heterogeneity |
| Cariprazine:Xanomeline-Trospium | Some concerns | Some concerns | No concerns | Major concerns | Major concerns | No concerns | Very low | Within-study bias, Reporting bias, Imprecision, Heterogeneity |
| Lumateperone:Xanomeline-Trospium | Some concerns | Some concerns | No concerns | Some concerns | Major concerns | No concerns | Low | Within-study bias, Reporting bias, Imprecision, Heterogeneity |

Table S9.2. for treatment discontinuation.

| **Comparison** | **Within Study Bias** | **Reporting Bias** | **Indirectness** | **Imprecision** | **Heterogeneity** | **Incoherence** | **Overall Confidence** | **Reason for downgrade** |
| --- | --- | --- | --- | --- | --- | --- | --- | --- |
| Asenapine:Placebo | Some concerns | Some concerns | No concerns | No concerns | No concerns | No concerns | Moderate | Within-study bias, Reporting bias |
| Cariprazine:Placebo | Some concerns | Some concerns | No concerns | No concerns | No concerns | No concerns | Moderate | Within-study bias, Reporting bias |
| Lumateperone:Placebo | Some concerns | Some concerns | No concerns | Major concerns | No concerns | No concerns | Low | Within-study bias, Reporting bias, Imprecision |
| Placebo:Xanomeline-Trospium | Some concerns | Some concerns | No concerns | No concerns | No concerns | No concerns | Moderate | Within-study bias, Reporting bias |
| Asenapine:Cariprazine | Some concerns | Some concerns | No concerns | No concerns | No concerns | No concerns | Moderate | Within-study bias, Reporting bias |
| Asenapine:Lumateperone | Some concerns | Some concerns | No concerns | Major concerns | No concerns | No concerns | Low | Within-study bias, Reporting bias, Imprecision |
| Asenapine:Xanomeline-Trospium | Some concerns | Some concerns | No concerns | Major concerns | No concerns | No concerns | Low | Within-study bias, Reporting bias, Imprecision |
| Cariprazine:Lumateperone | Some concerns | Some concerns | No concerns | Major concerns | No concerns | No concerns | Low | Within-study bias, Reporting bias, Imprecision |
| Cariprazine:Xanomeline-Trospium | Some concerns | Some concerns | No concerns | Major concerns | Major concerns | No concerns | Very low | Within-study bias, Reporting bias, Imprecision, Heterogeneity |
| Lumateperone:Xanomeline-Trospium | Some concerns | Some concerns | No concerns | Some concerns | Major concerns | No concerns | Low | Within-study bias, Reporting bias, Imprecision, Heterogeneity |

Table S9.3. for serious adverse events.

| **Comparison** | **Within Study Bias** | **Reporting Bias** | **Indirectness** | **Imprecision** | **Heterogeneity** | **Incoherence** | **Overall Confidence** | **Reason for downgrade** |
| --- | --- | --- | --- | --- | --- | --- | --- | --- |
| Asenapine:Placebo | Some concerns | Some concerns | No concerns | No concerns | Some concerns | No concerns | Low | Within-study bias, Reporting bias, Heterogeneity |
| Cariprazine:Placebo | Some concerns | Some concerns | No concerns | No concerns | Some concerns | No concerns | Low | Within-study bias, Reporting bias, Heterogeneity |
| Lumateperone:Placebo | Some concerns | Some concerns | No concerns | No concerns | Some concerns | No concerns | Low | Within-study bias, Reporting bias, Heterogeneity |
| Placebo:Xanomeline -Trospium | Some concerns | Some concerns | No concerns | Major concerns | Some concerns | No concerns | Low | Within-study bias, Reporting bias, Imprecision, Heterogeneity |
| Asenapine:Cariprazine | Some concerns | Some concerns | No concerns | No concerns | Some concerns | No concerns | Low | Within-study bias, Reporting bias, Heterogeneity |
| Asenapine:Lumateperone | Some concerns | Some concerns | No concerns | No concerns | Some concerns | No concerns | Low | Within-study bias, Reporting bias, Heterogeneity |
| Asenapine:Xanomeline -Trospium | Some concerns | Some concerns | No concerns | Some concerns | Some concerns | No concerns | Low | Within-study bias, Reporting bias, Imprecision, Heterogeneity |
| Cariprazine:Lumateperone | Some concerns | Some concerns | No concerns | Major concerns | Some concerns | No concerns | Low | Within-study bias, Reporting bias, Imprecision, Heterogeneity |
| Cariprazine:Xanomeline -Trospium | Some concerns | Some concerns | No concerns | Major concerns | Some concerns | No concerns | Low | Within-study bias, Reporting bias, Imprecision, Heterogeneity |
| Lumateperone:Xanomeline -Trospium | Some concerns | Some concerns | No concerns | No concerns | Some concerns | No concerns | Low | Within-study bias, Reporting bias, Heterogeneity |

Table S9.4. for ≥7 increase in body weight.

| **Comparison** | **Within Study Bias** | **Reporting Bias** | **Indirectness** | **Imprecision** | **Heterogeneity** | **Incoherence** | **Overall Confidence** | **Reason for downgrade** |
| --- | --- | --- | --- | --- | --- | --- | --- | --- |
| Asenapine:Placebo | Some concerns | Some concerns | No concerns | No concerns | No concerns | No concerns | Moderate | Within-study bias, Reporting bias |
| Cariprazine:Placebo | Some concerns | Some concerns | No concerns | No concerns | No concerns | No concerns | Moderate | Within-study bias, Reporting bias |
| Lumateperone:Placebo | Some concerns | Some concerns | No concerns | No concerns | No concerns | No concerns | Moderate | Within-study bias, Reporting bias |
| Placebo:Xanomeline-Trospium | Some concerns | Some concerns | No concerns | No concerns | No concerns | No concerns | Moderate | Within-study bias, Reporting bias |
| Asenapine:Cariprazine | Some concerns | Some concerns | No concerns | Some concerns | No concerns | No concerns | Low | Within-study bias, Reporting bias, Imprecision |
| Asenapine:Lumateperone | Some concerns | Some concerns | No concerns | Some concerns | No concerns | No concerns | Low | Within-study bias, Reporting bias, Imprecision |
| Asenapine:Xanomeline-Trospium | Some concerns | Some concerns | No concerns | Some concerns | No concerns | No concerns | Low | Within-study bias, Reporting bias, Imprecision |
| Cariprazine:Lumateperone | Some concerns | Some concerns | No concerns | Major concerns | No concerns | No concerns | Low | Within-study bias, Reporting bias, Imprecision |
| Cariprazine:Xanomeline-Trospium | Some concerns | Some concerns | No concerns | No concerns | No concerns | No concerns | Moderate | Within-study bias, Reporting bias |
| Lumateperone:Xanomeline-Trospium | Some concerns | Some concerns | No concerns | No concerns | No concerns | No concerns | Moderate | Within-study bias, Reporting bias |

Table S9.5. for akathisia.

| **Comparison** | **Within Study Bias** | **Reporting Bias** | **Indirectness** | **Imprecision** | **Heterogeneity** | **Incoherence** | **Overall Confidence** | **Reason for downgrade** |
| --- | --- | --- | --- | --- | --- | --- | --- | --- |
| Asenapine:Placebo | Some concerns | Some concerns | No concerns | No concerns | No concerns | No concerns | Moderate | Within-study bias, Reporting bias |
| Cariprazine:Placebo | Some concerns | Some concerns | No concerns | No concerns | No concerns | No concerns | Moderate | Within-study bias, Reporting bias |
| Lumateperone:Placebo | Some concerns | Some concerns | No concerns | Some concerns | No concerns | No concerns | Low | Within-study bias, Reporting bias, Imprecision |
| Placebo:Xanomeline-Trospium | Some concerns | Some concerns | No concerns | Major concerns | No concerns | No concerns | Low | Within-study bias, Reporting bias, Imprecision |
| Asenapine:Cariprazine | Some concerns | Some concerns | No concerns | Major concerns | No concerns | No concerns | Low | Within-study bias, Reporting bias, Imprecision |
| Asenapine:Lumateperone | Some concerns | Some concerns | No concerns | Major concerns | No concerns | No concerns | Low | Within-study bias, Reporting bias, Imprecision |
| Asenapine:Xanomeline-Trospium | Some concerns | Some concerns | No concerns | Major concerns | No concerns | No concerns | Low | Within-study bias, Reporting bias, Imprecision |
| Cariprazine:Lumateperone | Some concerns | Some concerns | No concerns | Some concerns | No concerns | No concerns | Low | Within-study bias, Reporting bias, Imprecision |
| Cariprazine:Xanomeline-Trospium | Some concerns | Some concerns | No concerns | Some concerns | No concerns | No concerns | Low | Within-study bias, Reporting bias, Imprecision |
| Lumateperone:Xanomeline-Trospium | Some concerns | Some concerns | No concerns | Major concerns | No concerns | No concerns | Low | Within-study bias, Reporting bias, Imprecision |

Table S9.6. for sedation and/or somnolence.

| **Comparison** | **Within Study Bias** | **Reporting Bias** | **Indirectness** | **Imprecision** | **Heterogeneity** | **Incoherence** | **Overall Confidence** | **Reason for downgrade** |
| --- | --- | --- | --- | --- | --- | --- | --- | --- |
| Asenapine:Placebo | Some concerns | Some concerns | No concerns | No concerns | Some concerns | No concerns | Low | Within-study bias, Reporting bias, Heterogeneity |
| Cariprazine:Placebo | Some concerns | Some concerns | No concerns | No concerns | Some concerns | No concerns | Low | Within-study bias, Reporting bias, Heterogeneity |
| Lumateperone:Placebo | Some concerns | Some concerns | No concerns | No concerns | Some concerns | No concerns | Low | Within-study bias, Reporting bias, Heterogeneity |
| Placebo:Xanomeline -Trospium | Some concerns | Some concerns | No concerns | Major concerns | Some concerns | No concerns | Low | Within-study bias, Reporting bias, Imprecision, Heterogeneity |
| Asenapine:Cariprazine | Some concerns | Some concerns | No concerns | No concerns | Some concerns | No concerns | Low | Within-study bias, Reporting bias, Heterogeneity |
| Asenapine:Lumateperone | Some concerns | Some concerns | No concerns | No concerns | Some concerns | No concerns | Low | Within-study bias, Reporting bias, Heterogeneity |
| Asenapine:Xanomeline -Trospium | Some concerns | Some concerns | No concerns | Some concerns | Some concerns | No concerns | Low | Within-study bias, Reporting bias, Imprecision, Heterogeneity |
| Cariprazine:Lumateperone | Some concerns | Some concerns | No concerns | Major concerns | Some concerns | No concerns | Low | Within-study bias, Reporting bias, Imprecision, Heterogeneity |
| Cariprazine:Xanomeline -Trospium | Some concerns | Some concerns | No concerns | Major concerns | Some concerns | No concerns | Low | Within-study bias, Reporting bias, Imprecision, Heterogeneity |
| Lumateperone:Xanomeline -Trospium | Some concerns | Some concerns | No concerns | No concerns | Some concerns | No concerns | Low | Within-study bias, Reporting bias, Heterogeneity |

Supplement S10**.** Sensitivity analysis.

Table S10.1 Leave-One-Out Analysis for PNASS total score.

| **Study Omitted** | **I^2^** | **Tau^2^** | **Interpretation** |
| --- | --- | --- | --- |
| Kinoshita, 2016 | 7.5% | 0.4598 | Effect Stable |
| Landbloom, 2016 | 0% | 0 | Effect Stable |
| Potkin, 2007 | 43.9% | 3.9943 | Effect Stable |
| Durgam, 2014 | 40.3% | 3.7458 | Effect Stable |
| Durgam, 2015 | 44.2% | 4.5268 | Effect Stable |
| Durgam, 2016 | 20.3% | 1.3909 | Effect Stable |
| Kane, 2015 | 42% | 4.0702 | Effect Stable |
| Correll, 2020 | 38.4% | 3.6082 | Effect Stable |
| Lieberman, 2016 | 38.4% | 3.6082 | Effect Stable |
| Brannan, 2021 | 39.9% | 3.7707 | Effect Stable |
| Kaul, 2024_a | 44.3% | 4.5418 | Effect Stable |
| Kaul, 2024_b | 41.1% | 4.0313 | Effect Stable |

Table S10.2 Leave-One-Out Analysis for treatment discontinuation.

| **Study Omitted** | **I^2^** | **Tau^2^** | **Interpretation** |
| --- | --- | --- | --- |
| Kinoshita, 2016 | 0% | 0 | Effect Stable |
| Landbloom, 2016 | 0% | 0 | Effect Stable |
| Potkin, 2007 | 0% | 0 | Effect Stable |
| Durgam, 2014 | 0% | 0 | Effect Stable |
| Durgam, 2015 | 0% | 0 | Effect Stable |
| Durgam, 2016 | 0% | 0 | Effect Stable |
| Kane, 2015 | 0% | 0 | Effect Stable |
| Correll, 2020 | 0% | 0 | Effect Stable |
| Lieberman, 2016 | 0% | 0 | Effect Stable |
| Brannan, 2021 | 0% | 0 | Effect Stable |
| Kaul, 2024_a | 0% | 0 | Effect Stable |
| Kaul, 2024_b | 0% | 0 | Effect Stable |

Table S10.3 Leave-One-Out Analysis for serious adverse events.

| **Study Omitted** | **I^2^** | **Tau^2^** | **Interpretation** |
| --- | --- | --- | --- |
| Kinoshita, 2016 | 0% | 0 | Effect Stable |
| Landbloom, 2016 | 0% | 0 | Effect Stable |
| Potkin, 2007 | 0% | 0 | Effect Stable |
| Durgam, 2014 | 0% | 0 | Effect Stable |
| Durgam, 2015 | 0% | 0 | Effect Stable |
| Durgam, 2016 | 0% | 0 | Effect Stable |
| Kane, 2015 | 0% | 0 | Effect Stable |
| Correll, 2020 | 0% | 0 | Effect Stable |
| Lieberman, 2016 | 0% | 0 | Effect Stable |
| Brannan, 2021 | 0% | 0 | Effect Stable |
| Kaul, 2024_a | 0% | 0 | Effect Stable |
| Kaul, 2024_b | 0% | 0 | Effect Stable |

Table S10.7 Leave-One-Out Analysis for ≥7 increase in body weight.

| **Study Omitted** | **I^2^** | **Tau^2^** | **Interpretation** |
| --- | --- | --- | --- |
| Kinoshita, 2016 | 0% | 0 | Effect Stable |
| Landbloom, 2016 | 0% | 0 | Effect Stable |
| Potkin, 2007 | 0% | 0 | Effect Stable |
| Durgam, 2014 | 0% | 0 | Effect Stable |
| Durgam, 2015 | 0% | 0 | Effect Stable |
| Kane, 2015 | 0% | 0 | Effect Stable |
| Correll, 2020 | 0% | 0 | Effect Stable |
| Lieberman, 2016 | 0% | 0 | Effect Stable |
| Brannan, 2021 | 0% | 0 | Effect Stable |
| Kaul, 2024_a | 0% | 0 | Effect Stable |
| Kaul, 2024_b | 0% | 0 | Effect Stable |

Table S10.4 Leave-One-Out Analysis for akathesia

| **Study Omitted** | **I^2^** | **Tau^2^** | **Interpretation** |
| --- | --- | --- | --- |
| Kinoshita, 2016 | 0% | 0 | Effect Stable |
| Landbloom, 2016 | 0% | 0 | Effect Stable |
| Durgam, 2014 | 0% | 0 | Effect Stable |
| Durgam, 2015 | 4.5% | 0.0225 | Effect Stable |
| Durgam, 2016 | 0% | 0 | Effect Stable |
| Kane, 2015 | 0% | 0 | Effect Stable |
| Correll, 2020 | 2.9% | 0.0111 | Effect Stable |
| Lieberman, 2016 | 2.9% | 0.0111 | Effect Stable |
| Brannan, 2021 | 1.5% | 0.0056 | Effect Stable |
| Kaul, 2024_a | 0% | 0 | Effect Stable |
| Kaul, 2024_b | 4.5% | 0.0168 | Effect Stable |

Table S10.5 Leave-One-Out Analysis for sedation and/or somnolence.

| **Study Omitted** | **I^2^** | **Tau^2^** | **Interpretation** |
| --- | --- | --- | --- |
| Kinoshita, 2016 | 0% | 0 | Effect Stable |
| Landbloom, 2016 | 37.7% | 0.1219 | Effect Stable |
| Potkin, 2007 | 0% | 0 | Effect Stable |
| Durgam, 2014 | 15.2% | 0.0394 | Effect Stable |
| Durgam, 2016 | 15.2% | 0.0394 | Effect Stable |
| Correll, 2020 | 35.2% | 0.1613 | Effect Stable |
| Lieberman, 2016 | 35.2% | 0.1613 | Effect Stable |
| Brannan, 2021 | 37.1% | 0.1193 | Effect Stable |
| Kaul, 2024_a | 37.4% | 0.1204 | Effect Stable |
| Kaul, 2024_b | 32.6% | 0.0905 | Effect Stable |

Table S10.6 Leave-One-Out Analysis for gastrointestinal side effects.

| **Study Omitted** | **I^2^** | **Tau^2^** | **Interpretation** |
| --- | --- | --- | --- |
| Kinoshita, 2016 | 70.5% | 0.1798 | Effect Stable |
| Landbloom, 2016 | 66.9% | 0.1267 | Effect Stable |
| Potkin, 2007 | 69% | 0.1676 | Effect Stable |
| Durgam, 2014 | 69.9% | 0.1640 | Effect Stable |
| Durgam, 2015 | 71.2% | 0.1633 | Effect Stable |
| Durgam, 2016 | 57.2% | 0.1075 | Effect Stable |
| Kane, 2015 | 63.8% | 0.1349 | Effect Stable |
| Correll, 2020 | 70.6% | 0.1510 | Effect Stable |
| Lieberman, 2016 | 70.6% | 0.1510 | Effect Stable |
| Brannan, 2021 | 69.1% | 0.1777 | Effect Stable |
| Kaul, 2024_a | 69.5% | 0.1891 | Effect Stable |
| Kaul, 2024_b | 49.2% | 0.0697 | Effect Stable |

Supplement S11. PRISMA check list.

| **Section and Topic** | **Item #** | **Checklist item** | **Location where item is reported** |
| --- | --- | --- | --- |
| **TITLE** | | |  |
| Title | 1 | Identify the report as a systematic review. | 1 |
| **ABSTRACT** | | |  |
| Abstract | 2 | See the PRISMA 2020 for Abstracts checklist. | 1 |
| **INTRODUCTION** | | |  |
| Rationale | 3 | Describe the rationale for the review in the context of existing knowledge. | 2 |
| Objectives | 4 | Provide an explicit statement of the objective(s) or question(s) the review addresses. | 2 |
| **METHODS** | | |  |
| Eligibility criteria | 5 | Specify the inclusion and exclusion criteria for the review and how studies were grouped for the syntheses. | 3 |
| Information sources | 6 | Specify all databases, registers, websites, organisations, reference lists and other sources searched or consulted to identify studies. Specify the date when each source was last searched or consulted. | 3 |
| Search strategy | 7 | Present the full search strategies for all databases, registers and websites, including any filters and limits used. | 3 |
| Selection process | 8 | Specify the methods used to decide whether a study met the inclusion criteria of the review, including how many reviewers screened each record and each report retrieved, whether they worked independently, and if applicable, details of automation tools used in the process. | 3 |
| Data collection process | 9 | Specify the methods used to collect data from reports, including how many reviewers collected data from each report, whether they worked independently, any processes for obtaining or confirming data from study investigators, and if applicable, details of automation tools used in the process. | 3 |
| Data items | 10a | List and define all outcomes for which data were sought. Specify whether all results that were compatible with each outcome domain in each study were sought (e.g. for all measures, time points, analyses), and if not, the methods used to decide which results to collect. | 4 |
|  | 10b | List and define all other variables for which data were sought (e.g. participant and intervention characteristics, funding sources). Describe any assumptions made about any missing or unclear information. | 4 |
| Study risk of bias assessment | 11 | Specify the methods used to assess risk of bias in the included studies, including details of the tool(s) used, how many reviewers assessed each study and whether they worked independently, and if applicable, details of automation tools used in the process. | 4 |
| Effect measures | 12 | Specify for each outcome the effect measure(s) (e.g. risk ratio, mean difference) used in the synthesis or presentation of results. | 4 |
| Synthesis methods | 13a | Describe the processes used to decide which studies were eligible for each synthesis (e.g. tabulating the study intervention characteristics and comparing against the planned groups for each synthesis (item #5)). | 4 |
|  | 13b | Describe any methods required to prepare the data for presentation or synthesis, such as handling of missing summary statistics, or data conversions. | 4 |
|  | 13c | Describe any methods used to tabulate or visually display results of individual studies and syntheses. | 4 |
|  | 13d | Describe any methods used to synthesize results and provide a rationale for the choice(s). If meta-analysis was performed, describe the model(s), method(s) to identify the presence and extent of statistical heterogeneity, and software package(s) used. | 4 |
|  | 13e | Describe any methods used to explore possible causes of heterogeneity among study results (e.g. subgroup analysis, meta-regression). | 4 |
|  | 13f | Describe any sensitivity analyses conducted to assess robustness of the synthesized results. | 4 |
| Reporting bias assessment | 14 | Describe any methods used to assess risk of bias due to missing results in a synthesis (arising from reporting biases). | 4 |
| Certainty assessment | 15 | Describe any methods used to assess certainty (or confidence) in the body of evidence for an outcome. | 4 |
| **RESULTS** | | |  |
| Study selection | 16a | Describe the results of the search and selection process, from the number of records identified in the search to the number of studies included in the review, ideally using a flow diagram. | 5, supplement,  figure 1 |
|  | 16b | Cite studies that might appear to meet the inclusion criteria, but which were excluded, and explain why they were excluded. | 5 |
| Study characteristics | 17 | Cite each included study and present its characteristics. | 5-7, supplement |
| Risk of bias in studies | 18 | Present assessments of risk of bias for each included study. | 5, supplement |
| Results of individual studies | 19 | For all outcomes, present, for each study: (a) summary statistics for each group (where appropriate) and (b) an effect estimate and its precision (e.g. confidence/credible interval), ideally using structured tables or plots. | 10-14 |
| Results of syntheses | 20a | For each synthesis, briefly summarise the characteristics and risk of bias among contributing studies. | 10-14 |
|  | 20b | Present results of all statistical syntheses conducted. If meta-analysis was done, present for each the summary estimate and its precision (e.g. confidence/credible interval) and measures of statistical heterogeneity. If comparing groups, describe the direction of the effect. | 10-14 |
|  | 20c | Present results of all investigations of possible causes of heterogeneity among study results. | 10-14 |
|  | 20d | Present results of all sensitivity analyses conducted to assess the robustness of the synthesized results. | 10-14, supplement |
| Reporting biases | 21 | Present assessments of risk of bias due to missing results (arising from reporting biases) for each synthesis assessed. | 5, supplement |
| Certainty of evidence | 22 | Present assessments of certainty (or confidence) in the body of evidence for each outcome assessed. | 5, supplement |
| **DISCUSSION** | | |  |
| Discussion | 23a | Provide a general interpretation of the results in the context of other evidence. | 14-17 |
|  | 23b | Discuss any limitations of the evidence included in the review. | 14-17 |
|  | 23c | Discuss any limitations of the review processes used. | 14-17 |
|  | 23d | Discuss implications of the results for practice, policy, and future research. | 14-17 |
| **OTHER INFORMATION** | | |  |
| Registration and protocol | 24a | Provide registration information for the review, including register name and registration number, or state that the review was not registered. | 3 |
|  | 24b | Indicate where the review protocol can be accessed, or state that a protocol was not prepared. | 3 |
|  | 24c | Describe and explain any amendments to information provided at registration or in the protocol. | 3 |
| Support | 25 | Describe sources of financial or non-financial support for the review, and the role of the funders or sponsors in the review. | 1 |
| Competing interests | 26 | Declare any competing interests of review authors. | 1 |
| Availability of data, code and other materials | 27 | Report which of the following are publicly available and where they can be found: template data collection forms; data extracted from included studies; data used for all analyses; analytic code; any other materials used in the review. | 1 |
